# Estimates of global SARS-CoV-2 infection exposure, infection morbidity, and infection mortality rates in 2020

**DOI:** 10.1016/j.gloepi.2021.100068

**Published:** 2021-11-23

**Authors:** Houssein H. Ayoub, Ghina R. Mumtaz, Shaheen Seedat, Monia Makhoul, Hiam Chemaitelly, Laith J. Abu-Raddad

**Affiliations:** a Mathematics Program, Department of Mathematics, Statistics, and Physics, College of Arts and Sciences, Qatar University, Doha, Qatar; b Department of Epidemiology and Population Health, American University of Beirut, Beirut, Lebanon; c Infectious Disease Epidemiology Group, Weill Cornell Medicine-Qatar, Cornell University, Doha, Qatar; d World Health Organization Collaborating Centre for Disease Epidemiology Analytics on HIV/AIDS, Sexually Transmitted Infections, and Viral Hepatitis, Weill Cornell Medicine–Qatar, Cornell University, Qatar Foundation – Education City, Doha, Qatar; e Department of Population Health Sciences, Weill Cornell Medicine, Cornell University, NY, New York, USA; f Department of Public Health, College of Health Sciences, QU Health, Qatar University, Doha, Qatar

**Keywords:** SARS-CoV-2, COVID, Coronavirus, Epidemiology, Severity, Fatality, Exposure, Prevalence

## Abstract

We aimed to estimate, albeit crudely and provisionally, national, regional, and global proportions of respective populations that have been infected with SARS-CoV-2 in the first year after the introduction of this virus into human circulation, and to assess infection morbidity and mortality rates, factoring both documented and undocumented infections. The estimates were generated by applying mathematical models to 159 countries and territories. The percentage of the world's population that has been infected as of 31 December 2020 was estimated at 12.56% (95% CI: 11.17–14.05%). It was lowest in the Western Pacific Region at 0.66% (95% CI: 0.59–0.75%) and highest in the Americas at 41.92% (95% CI: 37.95–46.09%). The global infection fatality rate was 10.73 (95% CI: 10.21–11.29) per 10,000 infections. Globally per 1000 infections, the infection acute-care bed hospitalization rate was 19.22 (95% CI: 18.73–19.51), the infection ICU bed hospitalization rate was 4.14 (95% CI: 4.10–4.18). If left unchecked with no vaccination and no other public health interventions, and assuming circulation of only wild-type variants and no variants of concern, the pandemic would eventually cause 8.18 million deaths (95% CI: 7.30–9.18), 163.67 million acute-care hospitalizations (95% CI: 148.12–179.51), and 33.01 million ICU hospitalizations (95% CI: 30.52–35.70), by the time the herd immunity threshold is reached at 60–70% infection exposure. The global population remained far below the herd immunity threshold by end of 2020. Global epidemiology reveals immense regional variation in infection exposure and morbidity and mortality rates.

## Introduction

The severe acute respiratory syndrome coronavirus 2 (SARS-CoV-2) pandemic continues to be a global health challenge with profound adverse consequences for human health, societies, and economies [1]. While our understanding of the epidemiology of SARS-CoV-2 infection and its Coronavirus Disease 2019 (COVID-19) disease burden has progressed in the year since it emerged, two questions remain largely unanswered.

1. “To what extent have populations of individual countries and the global population been infected by this virus in the first year of its introduction, regardless of whether those infections have been documented?” Infections include documented cases involving laboratory-confirmed diagnosis and undocumented asymptomatic or mild cases. While a growing number of serological surveys are being conducted to answer this question [[2], [3], [4], [5], [6], [7], [8], [9], [10]], the scope, scale, and geographic coverage of such studies remain limited.

2. “What are the *true* national and global COVID-19 morbidity and mortality rates in 2020, that is factoring all documented and undocumented infections?”

Recent scientific developments furnish an opportunity to provide answers, albeit crude approximations, to these questions. The growing number of serological surveys and analyses of national databases for this infection have shown that only about one in every ten infections have actually been diagnosed in 2020 [[2], [3], [4], [5], [6], [7], [8], [9], [10], [11]]. Moreover, a recent comprehensive analysis assessed the true infection morbidity and mortality rates for each age group, factoring both documented and undocumented infections [12].

Building on these developments, the objective of this study was to provide key provisional epidemiologic estimates nationally, regionally, and globally. These include estimates, for each country and territory with a population size >1 million, of the proportion of each population that has been already infected up to the end of 2020; estimates for the average incidence rate of this infection; and estimates for overall (total population) infection acute-care and intensive-care-unit (ICU) hospitalization rates, infection severity and criticality rates, and infection fatality rate. These estimates could have significant policy implications, more so in context of the mass vaccination campaigns ongoing worldwide in 2021. These estimates also clarify aspects of the epidemiology of SARS-CoV-2 in its first year of introduction; thus they can serve to benchmark estimates of the epidemiology of this virus in presence of the variants of concern that are dominating infection transmission in 2021.

## Materials and methods

### Definitions of epidemiologic outcome measures

Two criteria for classifying infection morbidity were used: one based on hospital admissions (acute-care or ICU) and one based on clinical presentations, as per the World Health Organization (WHO) classifications of disease severity (Table 1) [13]. While the two measures overlap, with severe cases typically admitted to acute-care beds and critical cases admitted to ICU beds, mild or moderately ill COVID-19 cases are sometimes hospitalized out of caution, because of other, concurrent indications, or as a form of isolation [12].

**Table 1:** Epidemiologic outcome measures estimated in this study for each country, by region, and globally.

Outcome measure	Definition	Interpretation
*Infection morbidity rates*		
*Morbidity based on hospitalization type*		
1. Infection acute-care bed hospitalization rate	Cumulative number of hospital admissions into acute-care beds over the cumulative number of infections, *documented and undocumented*	Proportion of infections that progress to acute-care bed hospital admission.
2. Infection ICU bed hospitalization rate	Cumulative number of hospital admissions into ICU beds over the cumulative number of infections, *documented and undocumented*	Proportion of infections that progress to ICU bed hospital admission.
3. Infection *total* hospitalization rate (measures 1 and 2 combined)	Cumulative number of hospital admissions into both acute-care and ICU beds over the cumulative number of infections, *documented and undocumented*	Proportion of infections that progress to acute-care or ICU bed hospital admission.

*Morbidity based on clinical presentation*^a^		
1. Infection severity rate	Cumulative number of COVID-19 severe infections⁎ over the cumulative number of infections, *documented and undocumented*	Proportion of infections that progress to become severe infections.
2. Infection criticality rate	Cumulative number of COVID-19 critical infections⁎ over the cumulative number of infections, *documented and undocumented*	Proportion of infections that progress to become critical infections.
3. Infection *total* severity rate (measures 4 and 5 combined)	Cumulative number of both COVID-19 severe or critical infections⁎ over the cumulative number of infections, *documented and undocumented*	Proportion of infections that progress to become severe or critical infections.

*Infection mortality rate*		
1. Infection fatality rate	Cumulative number of COVID-19 deaths over the cumulative number of infections, *documented and undocumented*	Proportion of infections that end in COVID-19 death.

*Infection occurrence metrics*		
1. Proportion of the population infected	Cumulative number of infections, *documented and undocumented*, over the total population size	Proportion of the population that has already infected sometime after the virus' introduction into the population.
2. Infection incidence rate (also known as force of infection or hazard rate of infection)	Number of new infections *documented and undocumented* per unit time (here week) over the size of the population that is still susceptible to the infection	Rate of new infections per person-weeks.

a Per World Health Organization infection severity classification [13].

Three types of outcomes were estimated nationally, regionally, and globally (Table 1) because of their public health relevance. The first two include *infection morbidity and mortality rates*, calculated as the cumulative number of a disease outcome (such as COVID-19 hospitalization or death) over the estimated cumulative number of infections, documented and undocumented. The first type includes the two sub-categories of morbidity, of hospitalization and clinical presentation. The third type includes two kinds of infection occurrence metrics, the proportion of the population that has been already infected and the infection incidence rate, both as of December 31, 2020.

### Estimations of epidemiologic outcome measures

#### Infection morbidity and mortality rates

The overall (total population) infection acute-care and ICU hospitalization rates, infection severity and criticality rates, and infection fatality rate were estimated for each country by applying the estimated age-stratified rates for these outcomes [12] to the population age-structure of each country. Age-stratified rates were based on a detailed analysis of the epidemic in Qatar [12] using data from a series of serological surveys [[8], [9], [10]] and extensive time-series and age-stratified data for PCR laboratory-confirmed infections, PCR testing positivity rate, antibody testing positivity rate, PCR surveys, daily hospital admissions in acute-care and ICU beds, hospital occupancy in acute-care and ICU beds, incidence of severe and critical infections, as per WHO classifications [13], and COVID-19 deaths as per WHO guidelines [14]. Qatar has one of the world's most extensive databases to document this epidemic and its toll at the national level [15], such that Qatar's epidemic has been one of the most thoroughly investigated and best characterized [[8], [9], [10], [11], [12],[15], [16], [17], [18], [19], [20], [21], [22], [23], [24], [25], [26], [27], [28], [29], [30], [31], [32], [33]].

It is presently unknown whether the infection morbidity and mortality rates for each age group (not for the total population) vary considerably from one country to another. These rates probably reflect primarily the basic biology of this infection more than the COVID-19 response or other factors in each country or population. The aim of the present study is to provide crude estimates for these rates in the total population of each country by factoring the population age structure, given the prominent role of age in the epidemiology of this pandemic [[34], [35], [36], [37]], while future studies investigate and elaborate possible variations in these rates across different countries and populations.

Infection morbidity and mortality rates were estimated for each country and territory with a population size >1 million, as of 2020. In total, estimates were generated for 159 countries and territories, virtually covering the world population [38]. Population sizes and demographic age-structures were extracted from the United Nations World Population Prospects database [38]. In each setting, rates were derived by weighting each rate in each age group by the proportion of the population in that age group, and then summing the contributions of all age groups.

#### Incidence rate and proportion of the population infected

Two methods were used to derive the proportion of the population infected in each country and the average incidence rate since onset of the epidemic as of the end of 2020. The final estimate for each of these measures was based on the average of the estimates of both methods, to minimize the effect of potential bias inherent in each method.

##### Reported COVID-19 deaths method

The first method was based on the reported number of COVID-19 deaths in each country, as per the WHO COVID-19 Dashboard [39]. The proportion of the population infected, irrespective of whether the infection was documented or undocumented, was estimated using the following expressions:

$$\mathrm{Proportion of the population infected}=\frac{\mathrm{Cumulative number of infections}}{\mathrm{Total population size}}$$

$$\mathrm{Cumulative number of infections}=\left(\frac{\mathrm{Number of reported deaths}}{\mathrm{Infection fatality rate}}\right)\times \left(\frac{\mathrm{HAQ}\;\mathrm{Index}}{\mathrm{Highest}\;\mathrm{HAQ}\;\mathrm{Index globaly}}\right)$$

Since COVID-19 mortality may be affected by access to and quality of healthcare, with higher mortality for inferior access and quality, this method adjusts for these factors by utilizing the Global Burden of Disease study's Healthcare Access and Quality (HAQ) Index for each country and territory [40]. The HAQ Index provides a score ranging between 0 and 100 [40].

The above expressions still require adjustment for the average time delay between onset of infection and COVID-19 death, estimated from studies in different countries at about 20 days [[41], [42], [43], [44]]. That adjustment was incorporated by assuming that the above estimated proportion of the population infected occurred 20 days earlier than the current time t. Then the average incidence rate of infection (*λ*), from the onset of the epidemic (at time t_0_) until the present time (time t), was derived using the expression:

$$\mathrm{Proportion of the population infected}\;\left(\left(t-20\;\mathrm{days}\right)-t_{0}\right)=1-e^{-\lambda \left(\left(t-20\;\mathrm{days}\right)-t_{0}\right)}$$

The time *t*_*0*_ was set as the day of the first reported COVID-19 case in each country [39]. The derived incidence rate was then used to estimate the proportion of the population infected at the time of this study, by applying the same expression, but at time *t* (that is at December 31, 2020), instead of *t* − 20 *days*.

##### Reported COVID-19 cases method

The second method was based on the reported cumulative number of laboratory-confirmed SARS-CoV-2 infections as of December 31, 2020, as reported in the WHO COVID-19 Dashboard [39]. The cumulative number of infections, documented and undocumented, was estimated using the following expression:

$$\mathrm{Cumulative number of infections}=\left(\frac{\mathrm{Cumulative number of documented infections}}{\mathrm{Infection detection rate}}\right)$$

The infection detection rate is defined as the cumulative number of documented infections, that is diagnosed and laboratory-confirmed, over the cumulative number of infections documented and undocumented. Serological surveys and extensive analyses have shown that only about one in every ten actual infections is ever diagnosed [[2], [3], [4], [5], [6], [7], [8], [9], [10]]. Given the quality estimate for the infection detection rate in the well-characterized epidemic of Qatar, based on a series of serological surveys [[8], [9], [10]] and analyses of national databases, a value of 11.1% (95% uncertainty interval: 10.8–11.3%) was assumed for the infection detection rate [11,12].

However, to account for variation in the quality of SARS-CoV-2 testing across countries, this estimate was adjusted using the HAQ Index [40]:

$$\mathrm{Infection detection rate in each country}=\left(\begin{array}{c} \mathrm{Infection detection rate}\\ \;\mathrm{in reference country} \end{array}\right)\times \left(\frac{\mathrm{HAQ}\;\mathrm{Index}}{\mathrm{HAQ}\;\mathrm{Index in reference country}}\right)$$

With the above expressions, and Qatar as the reference country, a second estimate was generated for each country of the proportion of the total population infected and the average incidence rate since epidemic onset.

### Uncertainty and sensitivity analyses

The 95% credible interval (CI) for each estimated epidemiologic outcome measure was derived by factoring the uncertainty interval of each variable used in the above equations and combining the uncertainties so as to generate the widest credible interval for each estimate.

The estimated proportion of the population infected using the reported COVID-19 cases method was assessed in a sensitivity analysis in which the adjustment using the HAQ ratio was taken to some power, to square and to square root, to provide a broad range of estimates for how this adjustment could affect the baseline estimates.

A second sensitivity analysis was conducted in which the proportion of the population infected using the reported COVID-19 cases method was assessed using a different input estimate for the infection detection rate, that for the United States (US) [45] instead of that for Qatar as the reference country, owing to the availability of a quality estimate for this rate in the US.

### Reporting of estimates

**Fig. 1:**
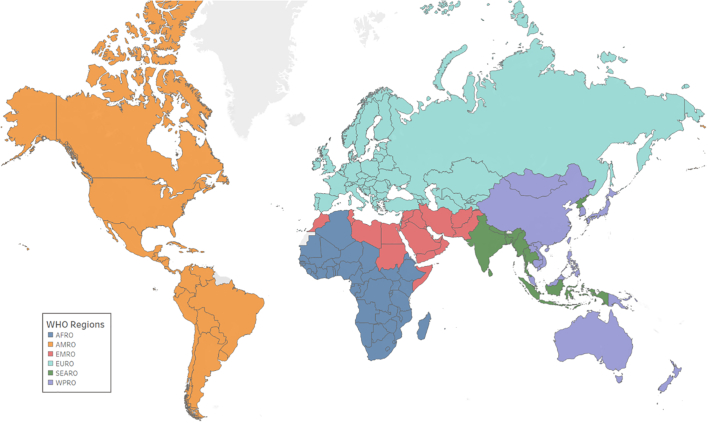
A global map of the World Health Organization (WHO) regions. The WHO regions include the African Region (AFRO) (blue), the Region of the Americas (AMRO) (orange), the Eastern Mediterranean Region (EMRO) (red), the European Region (EURO) (cyan), the South-East Asia Region (SEARO) (purple), and the Western Pacific Region (WPRO) (green) [46]. The map was generated using Tableau 10.1 software [71]. (For interpretation of the references to colour in this figure legend, the reader is referred to the web version of this article.)

The various estimated epidemiologic outcome measures and the 95% CI were reported by country or territory, regionally by WHO region, and globally. The WHO regions include the African Region (AFRO), the Region of the Americas (AMRO), the Eastern Mediterranean Region (EMRO), the European Region (EURO), the South-East Asia Region (SEARO), and the Western Pacific Region (WPRO) [46] (Fig. 1).

Mathematical modeling analyses were conducted in MATLAB R2019a (Boston/MA/USA) [47].

## Results

The various estimated outcome measures for each country and territory are listed in Tables S1-S6 in the Supporting Information. An overview of results by WHO region and globally is provided below.

**Fig. 2:**
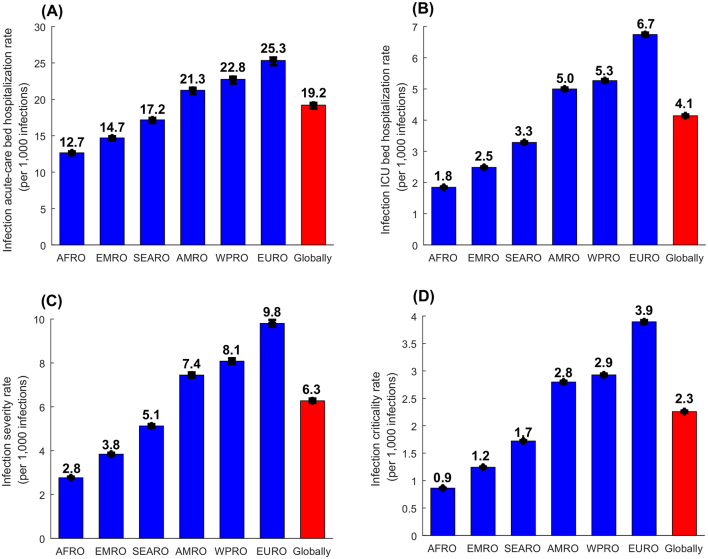
Estimated A) infection acute-care bed hospitalization rates, B) infection ICU bed hospitalization rates, C) infection severity rates, and D) infection criticality rates, across WHO regions and globally. WHO regions include the African Region (AFRO), the Eastern Mediterranean Region (EMRO), the South-East Asia Region (SEARO), the Region of the Americas (AMRO), the Western Pacific Region (WPRO), and the European Region (EURO) (Fig. 1). Classification of infection severity and criticality was per WHO infection severity criteria [13].

The estimated infection acute-care bed hospitalization rate, infection ICU bed hospitalization rate, infection severity rate, and infection criticality rate were lowest in AFRO and highest in EURO, with substantial variation across regions (Fig. 2). Globally, the infection acute-care bed hospitalization rate was 19.22 (95% CI: 18.73–19.51) per 1000 infections, the infection ICU bed hospitalization rate was 4.14 (95% CI: 4.10–4.18) per 1000 infections, the infection severity rate was 6.27 (95% CI: 6.18–6.37) per 1000 infections, and the infection criticality rate was 2.26 (95% CI: 2.24–2.28) per 1000 infections. Meanwhile, the infection total hospitalization rate was 23.36 (95% CI: 22.83–23.69) per 1000 infections and the infection total severity rate was 8.53 (95% CI: 8.42–8.65) per 1000 infections (Fig. 2).

**Fig. 3:**
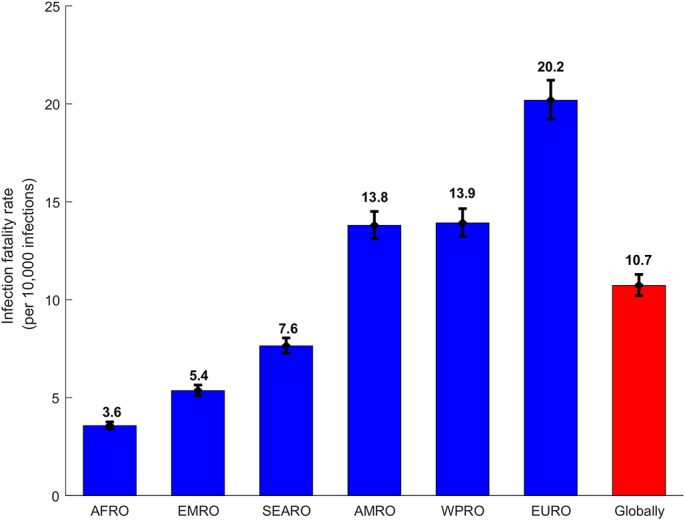
Estimated infection fatality rates, across WHO regions and globally. WHO regions include the African Region (AFRO), the Eastern Mediterranean Region (EMRO), the South-East Asia Region (SEARO), the Region of the Americas (AMRO), the Western Pacific Region (WPRO), and the European Region (EURO) (Fig. 1). Classification of COVID-19 mortality was per WHO criteria [14].

The estimated infection fatality rate per 10,000 infections, was lowest in AFRO at 3.57 (95% CI: 3.39–3.76), followed by EMRO at 5.40 (95% CI: 5.09–5.64), SEARO at 7.64 (95% CI: 7.26–8.05), AMRO at 13.80 (95% CI: 13.13–14.51), WPRO at 13.92 (95% CI: 13.24–14.65), and highest in EURO at 20.19 (95% CI: 19.23–21.21) (Fig. 3). Globally, the infection fatality rate was 10.73 (95% CI: 10.21–11.29) per 10,000 infections.

**Fig. 4:**
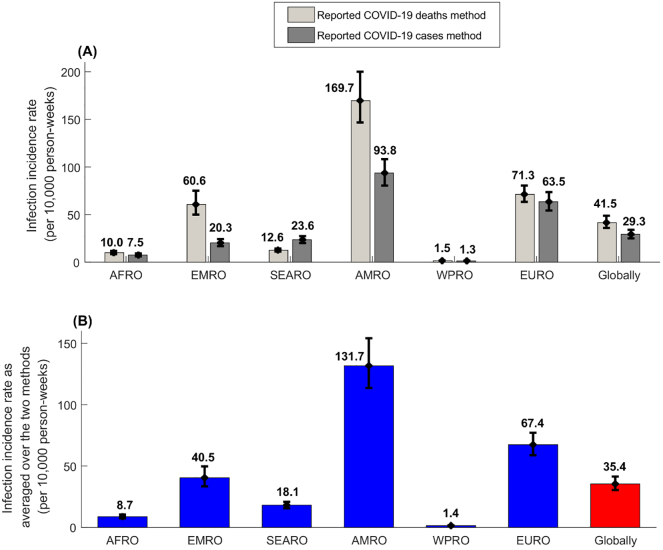
Estimated infection incidence rates across WHO regions and globally as of the end of 2020. A) Estimated average infection incidence rates since epidemic onset using the reported COVID-19 deaths method and using the reported COVID-19 cases method. B) Estimated infection incidence rates as averaged over the two methods. WHO regions include the African Region (AFRO), the Eastern Mediterranean Region (EMRO), the South-East Asia Region (SEARO), the Region of the Americas (AMRO), the Western Pacific Region (WPRO), and the European Region (EURO) (Fig. 1).

The estimated incidence rate by the end of 2020, across WHO regions and globally, using the reported deaths method was higher than that using the reported cases method, for all regions other than SEARO (Fig. 4A). The averaged incidence rate, per 10,000 person-weeks, was lowest in WPRO at 1.4 (95% CI: 1.2–1.6), followed by AFRO at 8.7 (95% CI: 7.2–10.5), SEARO at 18.1 (95% CI: 15.6–20.8), EMRO at 40.5 (95% CI: 33.4–49.7), EURO at 67.4 (95% CI: 58.8–77.1), and highest in AMRO at 131.7 (95% CI: 113.6–154.2) (Fig. 4B). Globally, the averaged incidence rate was 35.4 (95% CI: 30.5–41.4) per 10,000 person-weeks.

**Fig. 5:**
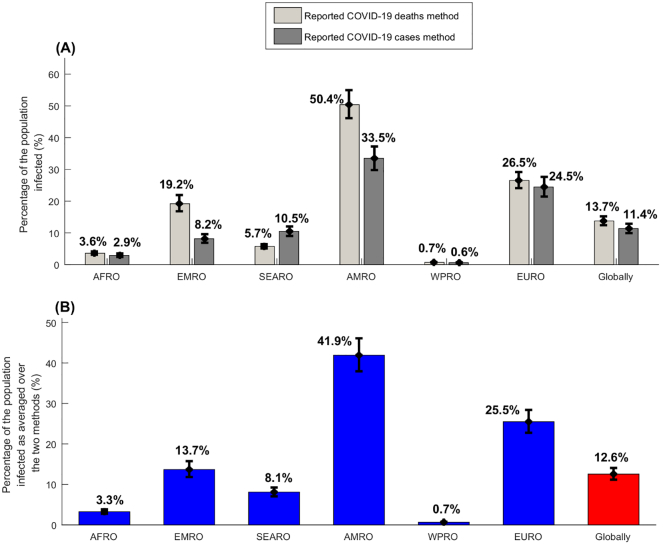
Estimated percentages of populations infected across WHO regions and globally as of the end of 2020. A) Estimated percentages of populations infected using the reported COVID-19 deaths method and the reported COVID-19 cases method. B) Estimated percentages of the population infected as averaged over the two methods. WHO regions include the African Region (AFRO), the Eastern Mediterranean Region (EMRO), the South-East Asia Region (SEARO), the Region of the Americas (AMRO), the Western Pacific Region (WPRO), and the European Region (EURO) (Fig. 1).

The estimated percentage of the population infected by the end of 2020, across WHO regions and globally was also higher using the reported deaths method than using the reported cases method, for all regions except SEARO (Fig. 5A). The averaged percentage of the population infected was lowest in WPRO at 0.66% (95% CI: 0.59–0.75%), followed by AFRO at 3.25% (95% CI: 2.75–3.84%), SEARO at 8.11% (95% CI: 7.05–9.24%), EMRO at 13.67% (95% CI: 11.83–15.75%), EURO at 25.48% (95% CI: 22.76–28.40%), and highest in AMRO at 41.91% (95% CI: 37.95–46.09%) (Fig. 5B). Globally, the averaged percentage of the population infected was estimated at 12.56% (95% CI: 11.17–14.05%).

As of December 31, 2020, 1.79 million COVID-19 deaths had been reported, but the percentage of the global population infected was estimated at only 12.56% (95% CI: 11.17–14.05%). If the global population is to reach herd immunity with no vaccination, conservatively estimated at 60–70% infection exposure for wild-type variants [10,48,49], then a cumulative total of 8.18 million COVID-19 deaths (95% CI: 7.30–9.18 million) would occur by the time herd immunity is reached. Also, by then, a cumulative total of 163.67 million acute-care hospitalizations (95% CI: 148.12–179.51), 33.01 million ICU hospitalizations (95% CI: 30.52–35.70), 50.23 million severe COVID-19 cases (95% CI: 46.24–54.67), and 17.62 million critical COVID-19 cases (95% CI: 16.36–18.97) would have occurred.

Figs. S1 and S2 show the results of the two sensitivity analyses. Both analyses generated similar results to the baseline analysis affirming that the global population remained far below the herd immunity threshold by end of 2020.

## Discussion

The above results suggest that only 13% of the world's population had been infected by SARS-CoV-2 by the end of 2020, even though an entire year had passed since the epidemic emerged in Wuhan, Hubei Province, China, in December 2019 [50,51]. This demonstrates that the overall global population remains far below the herd immunity threshold, estimated at 60–70% infection exposure (if not more with the new variants of concern) [10,48,49], and is still at risk of repeated epidemic waves of infection, with all that entails in terms of disease burden and social and economic disruption. This finding highlights the urgent need to accelerate COVID-19 vaccination to avert global expansion of this infection.

Though overall exposure to this infection remains relatively low as of the end of 2020, there are immense variations by region and country, and the Americas appear to have already reached ~40% exposure, over 60-fold higher than the Western Pacific Region (<1%). The average incidence rate experienced since epidemic onset up to the end of 2020 varies similarly and is highest in the Americas, at 132 per 10,000 person-weeks and lowest in the Western Pacific Region at only 1 per 10,000 person-weeks. These findings demonstrate strikingly high variability in the intensity of national epidemics during the first year since this infection's introduction. It remains to be seen whether this variability reflects different national responses to the epidemics, and/or clinical or biological cofactors that make some populations more affected than others.

Even though the same *age-stratified* infection morbidity and mortality rates were used in generating estimates for all countries, total-population morbidity and mortality rates varied hugely among countries and by region, only because of differences in population age structures. For instance, the infection fatality rate in the European Region of 20 per 10,000 infections was nearly 6-fold higher than that in the African Region at <4 per 10,000 infections. Similarly, infection hospitalization and severity rates varied enormously. These findings may explain apparent variability in the severity of this infection across countries and regions, and suggest that the disease burden could be substantially lower in countries with younger demographics, such as the African or Eastern Mediterranean Regions, as suggested earlier [35].

Notwithstanding this global variability, the above results corroborate the vast disease burden that this infection can cause. Nearly two million deaths have been confirmed worldwide as of December 31, 2020 [39], though only 13% of the global population has been infected. If we were to adopt today a herd immunity approach to dealing with this pandemic worldwide, that is, achieving the herd immunity threshold without vaccination, the pandemic would cause a total of 8 million COVID-19 deaths, 68 million COVID-19 severe and critical disease cases, and 197 million hospitalizations. These estimates would be even considerably higher in presence of variants of concern and their higher severity and infectiousness [33,[52], [53], [54], [55]]. These findings affirm the wisdom of epidemic suppression approaches adopted in most countries to tackle their respective epidemics [56].

Despite the high potential disease burden, the above-estimated infection morbidity and mortality rates are still substantially lower than those estimated earlier in the epidemic [6,[57], [58], [59], [60], [61]]. Globally, out of every 10,000 infections, only 11 would result in COVID-19 deaths. Out of every 1000 infections, only 6 would be severe and only 2 would be critical per WHO classification [13]. Nineteen would be hospitalized in an acute-care bed and 4 in an ICU bed.

Different methods have been used to estimate the proportion of the population infected and the infection fatality rate in different countries [45,62,63]. Our estimates are overall within the range of other estimates that have been completed for different countries [45,[63], [64], [65]]. To our knowledge, this study is the first to provide such national, regional, and global estimates, factoring both documented and undocumented infections in 2020, that is right before the onset of the global mass vaccination drive.

This study has limitations. In essence, it was based on age-stratified infection morbidity and mortality rates and the infection detection rate, estimated for a well-characterized and thoroughly investigated national epidemic, in which about half the population has already been infected [[8], [9], [10], [11], [12],[15], [16], [17], [18], [19], [20], [21], [22], [23]]. While the epidemic of Qatar is well-understood [[8], [9], [10], [11], [12],[15], [16], [17], [18], [19], [20], [21], [22], [23]], the extent to which fundamental infection metrics estimated with precision for one country, even if they are primarily determined by the basic biology of this infection, can be extrapolated to other countries, remains unknown.

It is also reasonable that these metrics could be affected by myriad factors, such as clinical or biological variations in human populations and circulating viral strains, the nature of COVID-19 responses, coverage of SARS-CoV-2 testing, quality and validity of reporting of cases and deaths, and the definition of reporting for COVID-19 cases and deaths. For example, in the US, COVID-19 deaths are reported if COVID-19 is listed in the death certificate as either an underlying or contributing cause of death [[66], [67], [68]], that is with differences compared to the WHO definition [14]. Reporting of COVID-19 deaths can also be affected by other factors such as filling and coding quality of death certificates and the location of death (hospital, nursing home, or at home) [66]. The underestimation of daily numbers of COVID-19 cases and deaths can also vary substantially by countries [69], which may confound the presented estimates. For instance, the ratio of the true mortality relative to the reported mortality in resource-limited settings such as sub-Saharan Africa could be higher than accounted for here. The ratio in sub-Saharan Africa has been estimated to range between 1.6 and 4.1 [70], suggesting that the total number of COVID-19 deaths in this region could be substantially underestimated.

Infection exposure is likely overestimated for countries with higher testing coverage and underestimated for countries with lower coverage. However, to make these estimates as realistic as possible, we used two independent methods with different input data to estimate infection exposure, to minimize the effect of any potential bias in either method or its data input. We also adjusted estimates for variation in healthcare access and quality by utilizing the Global Burden of Disease study's Healthcare Access and Quality Index for each country [40]. We further conducted sensitivity analyses whose results supported similar findings (Figs. S1-S2). Still, the provided estimates should be seen as provisional, crude estimates for the purpose of providing a broad understanding of the global epidemiology of this infection in its first year and to guide the global COVID-19 response.

## Conclusions

Albeit crudely and provisionally, we estimate that only 13% of the global population had been infected with SARS-CoV-2 by the end of 2020, suggesting that the world's population remains far below the herd immunity threshold and at risk of repeated epidemic waves of infection. Nevertheless, global epidemiology demonstrates immense regional variation in both infection exposure and SARS-CoV-2 morbidity and mortality rates. While the pandemic's expansion in nations with young populations could lead to a relatively milder disease burden than current expectations, this infection with its emerging variants of concern has the potential to easily cause ten million COVID-19 deaths and 200 million hospitalizations worldwide, if its transmission is left unchecked and vaccination scale-up continues to lag far behind global needs.

## Data availability

All data generated or analyzed during this study are included in this article and its Supplementary Information file.

## Funding

Developed mathematical models were made possible by NPRP grant number 9-040-3-008 (Principal investigator: L.J.A.) and NPRP grant number 12S-0216-190094 (Principal investigator: L.J.A.) from the 10.13039/100008982Qatar National Research Fund (a member of Qatar Foundation; https://www.qnrf.org). G.R.M. acknowledges support by 10.13039/100014013UK Research and Innovation as part of the Global Challenges Research Fund, grant number ES/P010873/1. The statements made herein are solely the responsibility of the authors. The funders had no role in study design, data collection and analysis, decision to publish, or preparation of the manuscript.

## Author contributions

HHA constructed and parameterized the mathematical model, conducted the mathematical modeling analyses, and co-wrote the first draft of the manuscript. HC and GM contributed to the parameterization of the model. LJA conceived and led the design of the study and model, conduct of analyses, and co-wrote the first draft of the manuscript. All authors contributed to discussions and interpretation of the results and to the writing of the manuscript. All authors have read and approved the final manuscript.

## Declaration of Competing Interest

We declare no competing interests.

## Appendix A Supplementary data

Supplementary material. Image 1
