# Supplementary material for: Estimates of global SARS-CoV-2 infection exposure, infection morbidity, and infection mortality rates in 2020
Source: Glob Epidemiol. 2021 Nov 23;3:100068. doi: 10.1016/j.gloepi.2021.100068 (PMC8609676; doi:10.1016/j.gloepi.2021.100068)
Supplement: Supplementary file 1 — Supplementary material. [file mmc1.docx]

**Supporting Information**

Estimates for the global SARS-CoV-2 infection exposure and infection morbidity and mortality rates in 2020

**Table S1:** Estimates for key SARS-CoV-2 epidemiologic indicators for countries and territories with a population of at least one million [[1](#_ENREF_1)] in the WHO African Region (AFRO). Classification of infection severity and criticality was per WHO infection severity classifications [[2](#_ENREF_2)]. Classification of COVID-19 death was also per WHO guidelines [[3](#_ENREF_3)].

| **African Region** | **Total population [**[**1**](#_ENREF_1)**]**^a^ | **HAQ Index [**[**4**](#_ENREF_4)**]**^b^ | **Infection acute-care bed hospitalization rate (per 1,000 infections)** | **Infection ICU bed hospitalization rate (per 1,000 infections)** | **Infection severity rate**  **(per 1,000 infections)** | **Infection criticality rate**  **(per 1,000 infections)** | **Infection fatality rate**  **(per 10,000 infections)** | **Infection detection rate (%)** | **Incidence rate since epidemic onset (per 10,000 person-weeks)**^c^ | **Proportion of the population infected (%)**^d^ |
| --- | --- | --- | --- | --- | --- | --- | --- | --- | --- | --- |
| **Country** | **Thousands** | **N (95% CI)** | **N (95% CI)** | **N (95% CI)** | **N (95% CI)** | **N (95% CI)** | **N (95% CI)** | **N (95% CI)** | **N (95% CI)** | **N (95% CI)** |
| Algeria | 43,851,043 | 63.1 (59.4-66.4) | 16.7 (16.3-16.9) | 3.2 (3.2-3.2) | 4.9 (4.8-5.0) | 1.7 (1.7-1.7) | 7.8 (7.4-8.2) | 8.5 (7.4-9.9) | 9.6 (8.4-10.9) | 4.1 (3.6-4.7) |
| Angola | 32,866,268 | 33.4 (25.5-40.4) | 11.6 (11.3-11.8) | 1.5 (1.5-1.5) | 2.2 (2.2-2.2) | 0.7 (0.7-0.7) | 2.6 (2.5-2.8) | 4.5 (3.2-6.0) | 3.6 (2.6-4.8) | 1.4 (1.1-2.0) |
| Benin | 12,123,198 | 30.8 (27.8-34.0) | 12.7 (12.4-12.9) | 1.9 (1.9-1.9) | 2.8 (2.8-2.9) | 0.9 (0.9-0.9) | 3.8 (3.6-4.0) | 4.2 (3.5-5.1) | 1.2 (1.0-1.4) | 0.5 (0.4-0.6) |
| [Botswana](http://www.who.int/countries/bwa/en/) | 2,351,625 | 51.5 (40.8-69.2) | 14.4 (14.1-14.6) | 2.4 (2.4-2.4) | 3.7 (3.6-3.8) | 1.2 (1.2-1.2) | 5.0 (4.8-5.3) | 7.0 (5.1-10.3) | 13.9 (9.5-19.6) | 5.3 (3.6-7.3) |
| [Burkina Faso](http://www.who.int/countries/bfa/en/) | 20,903,278 | 30.1 (27.0-33.3) | 11.8 (11.5-12.0) | 1.6 (1.6-1.6) | 2.3 (2.3-2.4) | 0.7 (0.7-0.7) | 2.8 (2.6-2.9) | 4.1 (3.4-5.0) | 1.5 (1.2-1.8) | 0.6 (0.5-0.7) |
| Burundi | 11,890,781 | 27.4 (23.1-32.1) | 11.7 (11.4-11.8) | 1.5 (1.5-1.5) | 2.2 (2.2-2.3) | 0.7 (0.7-0.7) | 2.7 (2.5-2.8) | 3.7 (2.9-4.8) | 0.3 (0.2-0.3) | 0.1 (0.1-0.1) |
| Cameroon | 26,545,864 | 31.9 (26.9-37.5) | 12.2 (11.9-12.3) | 1.7 (1.7-1.7) | 2.5 (2.5-2.6) | 0.8 (0.8-0.8) | 3.1 (2.9-3.2) | 4.3 (3.4-5.6) | 5.0 (3.9-6.4) | 2.1 (1.7-2.7) |
| Cent. Afr. Rep. | 4,829,764 | 18.6 (13.1-24.4) | 12.1 (11.8-12.3) | 1.7 (1.7-1.7) | 2.5 (2.4-2.5) | 0.8 (0.8-0.8) | 3.2 (3.0-3.4) | 2.5 (1.6-3.6) | 6.0 (4.1-9.2) | 2.5 (1.7-3.7) |
| Chad | 16,425,859 | 25.4 (21.9-29.0) | 11.6 (11.3-11.8) | 1.5 (1.5-1.6) | 2.2 (2.2-2.3) | 0.7 (0.7-0.7) | 2.8 (2.7-2.9) | 3.4 (2.7-4.3) | 1.2 (1.0-1.5) | 0.5 (0.4-0.6) |
| Congo | 5,518,092 | 34.1 (28.4-40.4) | 12.6 (12.3-12.8) | 1.8 (1.8-1.8) | 2.7 (2.7-2.8) | 0.8 (0.8-0.8) | 3.2 (3.0-3.4) | 4.6 (3.5-6.0) | 5.6 (4.3-7.2) | 2.3 (1.8-2.9) |
| [Côte d'Ivoire](http://www.who.int/countries/civ/en/) | 26,378,275 | 27.3 (24.2-31.1) | 12.4 (12.1-12.6) | 1.7 (1.7-1.8) | 2.6 (2.6-2.7) | 0.8 (0.8-0.8) | 3.2 (3.0-3.4) | 3.7 (3.0-4.6) | 3.3 (2.7-4.1) | 1.4 (1.1-1.7) |
| [D. Rep. Congo](http://www.who.int/countries/cod/en/) | 89,561,404 | 29.6 (25.7-33.7) | 12.3 (12.0-12.5) | 1.8 (1.7-1.8) | 2.6 (2.5-2.6) | 0.8 (0.8-0.8) | 3.4 (3.3-3.6) | 4.0 (3.2-5.0) | 1.3 (1.1-1.6) | 0.6 (0.4-0.7) |
| [Equ. Guinea](http://www.who.int/countries/gnq/en/) | 1,402,985 | 49.3 (38.3-62.0) | 11.9 (11.6-12.0) | 1.6 (1.5-1.6) | 2.4 (2.4-2.4) | 0.7 (0.7-0.7) | 2.7 (2.5-2.8) | 6.7 (4.8-9.2) | 22.9 (16.4-31.8) | 9.0 (6.6-12.3) |
| [Eritrea](http://www.who.int/countries/eri/en/) | 3,546,427 | 27.6 (23.7-31.3) | 13.6 (13.2-13.8) | 2.2 (2.2-2.3) | 3.3 (3.2-3.3) | 1.1 (1.1-1.1) | 5.0 (4.8-5.3) | 3.7 (3.0-4.7) | 1.2 (0.9-1.5) | 0.5 (0.4-0.6) |
| [Ethiopia](http://www.who.int/countries/eth/en/) | 114,963,583 | 28.1 (24.3-32.2) | 12.8 (12.5-13.0) | 2.0 (1.9-2.0) | 2.9 (2.8-2.9) | 0.9 (0.9-0.9) | 4.1 (3.8-4.3) | 3.8 (3.0-4.8) | 5.0 (4.0-6.2) | 2.1 (1.6-2.6) |
| Eswatini | 1,160,164 | 40.5 (30.4-52.2) | 13.1 (12.8-13.3) | 2.1 (2.1-2.1) | 3.1 (3.0-3.1) | 1.0 (1.0-1.0) | 4.6 (4.3-4.8) | 5.5 (3.8-7.8) | 39.1 (26.8-57.1) | 15.0 (10.6-21.2) |
| [Gabon](http://www.who.int/countries/gab/en/) | 2,225,728 | 40.4 (35.0-46.1) | 13.3 (13.0-13.5) | 2.1 (2.0-2.1) | 3.1 (3.1-3.2) | 1.0 (1.0-1.0) | 4.1 (3.9-4.3) | 5.5 (4.4-6.9) | 13.6 (10.8-17.1) | 5.5 (4.4-6.8) |
| [Gambia](http://www.who.int/countries/gmb/en/) | 2,416,664 | 35.7 (32.3-39.3) | 11.9 (11.6-12.1) | 1.6 (1.6-1.6) | 2.4 (2.3-2.4) | 0.7 (0.7-0.7) | 2.9 (2.7-3.1) | 4.8 (4.0-5.9) | 12.9 (10.7-15.4) | 5.1 (4.3-6.1) |
| Ghana | 31,072,945 | 39.3 (36.0-43.4) | 13.3 (13.0-13.5) | 2.0 (2.0-2.0) | 3.1 (3.0-3.1) | 0.9 (0.9-0.9) | 3.7 (3.5-3.9) | 5.3 (4.5-6.5) | 5.6 (4.6-6.6) | 2.3 (1.9-2.7) |
| Guinea | 13,132,792 | 26.4 (22.6-30.2) | 12.2 (11.9-12.4) | 1.7 (1.7-1.7) | 2.5 (2.5-2.6) | 0.8 (0.8-0.8) | 3.2 (3.1-3.4) | 3.6 (2.8-4.5) | 4.2 (3.3-5.3) | 1.7 (1.4-2.2) |
| Guinea-Bissau | 1,967,998 | 23.4 (20.2-26.8) | 12.3 (12.0-12.5) | 1.7 (1.7-1.7) | 2.6 (2.5-2.6) | 0.8 (0.8-0.8) | 3.1 (2.9-3.3) | 3.2 (2.5-4.0) | 7.4 (5.9-9.3) | 2.9 (2.3-3.7) |
| Kenya | 53,771,300 | 39.5 (35.0-43.9) | 12.2 (11.9-12.4) | 1.6 (1.6-1.7) | 2.5 (2.5-2.6) | 0.7 (0.7-0.7) | 2.9 (2.7-3.0) | 5.3 (4.4-6.5) | 9.9 (8.1-12.0) | 4.0 (3.3-4.9) |
| Lesotho | 2,142,252 | 32.0 (24.6-40.3) | 14.7 (14.3-14.9) | 2.5 (2.5-2.6) | 3.9 (3.8-3.9) | 1.3 (1.3-1.3) | 5.7 (5.4-6.0) | 4.3 (3.1-6.0) | 6.6 (4.7-9.2) | 2.1 (1.5-3.0) |
| [Liberia](http://www.who.int/countries/lbr/en/) | 5,057,677 | 32.2 (29.3-35.4) | 12.9 (12.6-13.1) | 1.9 (1.9-2.0) | 2.9 (2.9-2.9) | 0.9 (0.9-0.9) | 3.8 (3.6-4.0) | 4.4 (3.7-5.3) | 2.9 (2.4-3.4) | 1.2 (1.0-1.4) |
| Madagascar | 27,691,019 | 29.6 (24.3-35.1) | 12.7 (12.4-12.9) | 1.8 (1.8-1.9) | 2.8 (2.7-2.8) | 0.9 (0.9-0.9) | 3.5 (3.4-3.7) | 4.0 (3.0-5.2) | 3.1 (2.3-4.0) | 1.2 (0.9-1.6) |
| Malawi | 19,129,955 | 32.2 (26.9-38.1) | 11.9 (11.6-12.1) | 1.6 (1.6-1.7) | 2.4 (2.4-2.4) | 0.7 (0.7-0.7) | 3.0 (2.9-3.2) | 4.4 (3.4-5.7) | 2.5 (1.9-3.2) | 1.0 (0.8-1.2) |
| Mali | 20,250,834 | 34.9 (29.9-40.1) | 11.7 (11.4-11.9) | 1.5 (1.5-1.6) | 2.2 (2.2-2.3) | 0.7 (0.7-0.7) | 2.7 (2.6-2.9) | 4.7 (3.7-6.0) | 3.3 (2.6-4.1) | 1.3 (1.0-1.6) |
| [Mauritania](http://www.who.int/countries/mrt/en/) | 4,649,660 | 40.6 (35.0-47.5) | 12.9 (12.6-13.1) | 1.9 (1.9-1.9) | 2.9 (2.8-2.9) | 0.9 (0.9-0.9) | 3.7 (3.5-3.9) | 5.5 (4.4-7.1) | 17.2 (13.6-21.9) | 6.9 (5.5-8.7) |
| [Mauritius](http://www.who.int/countries/mus/en/) | 1,271,767 | 68.7 (65.5-71.9) | 23.0 (22.4-23.4) | 5.3 (5.3-5.4) | 8.2 (8.1-8.3) | 3.0 (2.9-3.0) | 13.9 (13.3-14.7) | 9.3 (8.2-10.7) | 1.1 (0.9-1.2) | 0.4 (0.4-0.5) |
| [Mozambique](http://www.who.int/countries/moz/en/) | 31,255,435 | 30.0 (25.3-35.0) | 12.1 (11.8-12.3) | 1.7 (1.7-1.7) | 2.5 (2.4-2.5) | 0.8 (0.8-0.8) | 3.2 (3.1-3.4) | 4.1 (3.2-5.2) | 2.5 (1.9-3.2) | 1.0 (0.8-1.3) |
| [Namibia](http://www.who.int/countries/nam/en/) | 2,540,916 | 44.6 (36.4-56.2) | 13.3 (12.9-13.5) | 2.1 (2.0-2.1) | 3.1 (3.1-3.2) | 1.0 (1.0-1.0) | 4.2 (4.0-4.4) | 6.0 (4.5-8.4) | 31.1 (22.5-42.7) | 12.1 (8.9-16.2) |
| [Niger](http://www.who.int/countries/ner/en/) | 24,206,636 | 28.4 (23.9-33.1) | 11.8 (11.5-11.9) | 1.6 (1.6-1.6) | 2.3 (2.2-2.3) | 0.7 (0.7-0.7) | 2.9 (2.7-3.0) | 3.8 (3.0-4.9) | 0.9 (0.7-1.2) | 0.4 (0.3-0.5) |
| [Nigeria](http://www.who.int/countries/nga/en/) | 206,139,587 | 41.9 (37.2-47.3) | 12.4 (12.1-12.6) | 1.7 (1.7-1.7) | 2.6 (2.5-2.6) | 0.8 (0.8-0.8) | 3.0 (2.9-3.2) | 5.7 (4.6-7.1) | 1.9 (1.6-2.3) | 0.8 (0.7-1.0) |
| [Rwanda](http://www.who.int/countries/rwa/en/) | 12,952,209 | 36.0 (31.6-40.5) | 12.8 (12.5-13.0) | 1.9 (1.8-1.9) | 2.8 (2.8-2.9) | 0.9 (0.9-0.9) | 3.5 (3.3-3.7) | 4.9 (3.9-6.0) | 2.5 (2.0-3.1) | 1.0 (0.8-1.3) |
| [Senegal](http://www.who.int/countries/sen/en/) | 16,743,930 | 31.1 (28.3-33.8) | 12.5 (12.2-12.7) | 1.8 (1.8-1.8) | 2.7 (2.6-2.7) | 0.8 (0.8-0.8) | 3.5 (3.3-3.7) | 4.2 (3.5-5.0) | 5.9 (5.0-7.0) | 2.5 (2.1-3.0) |
| [Sierra Leone](http://www.who.int/countries/sle/en/) | 7,976,985 | 31.0 (27.4-34.5) | 12.5 (12.2-12.7) | 1.8 (1.8-1.8) | 2.7 (2.6-2.7) | 0.8 (0.8-0.8) | 3.4 (3.2-3.5) | 4.2 (3.4-5.1) | 2.2 (1.8-2.7) | 0.9 (0.7-1.1) |
| [South Africa](http://www.who.int/countries/zaf/en/) | 59,308,690 | 49.7 (47.2-52.4) | 15.8 (15.4-16.1) | 2.8 (2.8-2.8) | 4.4 (4.3-4.5) | 1.4 (1.4-1.4) | 6.2 (5.9-6.5) | 6.7 (5.9-7.8) | 97.3 (83.1-114.3) | 33.7 (29.7-38.2) |
| [South Sudan](http://www.who.int/countries/ssd/en/) | 11,193,729 | 26.8 (21.0-33.1) | 12.8 (12.5-13.0) | 1.9 (1.9-2.0) | 2.9 (2.8-2.9) | 0.9 (0.9-0.9) | 3.9 (3.7-4.1) | 3.6 (2.6-4.9) | 1.7 (1.2-2.3) | 0.7 (0.5-0.9) |
| [Togo](http://www.who.int/countries/tgo/en/) | 8,278,737 | 32.0 (28.7-35.6) | 12.6 (12.3-12.8) | 1.8 (1.8-1.8) | 2.7 (2.7-2.8) | 0.8 (0.8-0.8) | 3.3 (3.1-3.4) | 4.3 (3.6-5.3) | 2.2 (1.8-2.7) | 0.9 (0.8-1.1) |
| [Uganda](http://www.who.int/countries/uga/en/) | 45,741,000 | 31.4 (27.2-35.6) | 11.2 (10.9-11.4) | 1.4 (1.4-1.4) | 2.0 (2.0-2.0) | 0.6 (0.6-0.6) | 2.3 (2.2-2.4) | 4.2 (3.4-5.3) | 3.2 (2.6-4.0) | 1.3 (1.1-1.6) |
| [Tanzania](http://www.who.int/countries/tza/en/) | 59,734,213 | 33.9 (30.0-38.4) | 12.1 (11.8-12.3) | 1.7 (1.6-1.7) | 2.5 (2.4-2.5) | 0.8 (0.7-0.8) | 3.0 (2.9-3.2) | 4.6 (3.7-5.7) | 0.1 (0.1-0.1) | 0.0 (0.0-0.0) |
| [Zambia](http://www.who.int/countries/zmb/en/) | 18,383,956 | 29.0 (23.0-35.4) | 11.4 (11.1-11.6) | 1.5 (1.4-1.5) | 2.1 (2.1-2.2) | 0.6 (0.6-0.6) | 2.5 (2.4-2.6) | 3.9 (2.9-5.3) | 6.8 (5.1-9.2) | 2.8 (2.1-3.7) |
| [Zimbabwe](http://www.who.int/countries/zwe/en/) | 14,862,927 | 31.2 (25.8-37.0) | 12.3 (12.0-12.5) | 1.8 (1.7-1.8) | 2.6 (2.6-2.7) | 0.8 (0.8-0.8) | 3.4 (3.3-3.6) | 4.2 (3.2-5.5) | 5.7 (4.4-7.4) | 2.3 (1.8-3.0) |
| **AFRO** | **1,118,418,151** | **35.6 (31.3-40.2)** | **12.7 (12.3-12.8)** | **1.8 (1.8-1.9)** | **2.8 (2.7-2.8)** | **0.9 (0.9-0.9)** | **3.6 (3.4-3.8)** | **4.8 (3.9-6.0)** | **8.7 (7.2-10.5)** | **3.3 (2.8-3.8)** |

Cent Afr Rep, Central African Republic. CI, credible interval. D Rep Congo, [Democratic Republic of the Congo](http://www.who.int/countries/cod/en/). [Equ Guinea](http://www.who.int/countries/gnq/en/), [Equatorial Guinea](http://www.who.int/countries/gnq/en/). HAQ, Healthcare Access and Quality. N, number. [Tanzania](http://www.who.int/countries/tza/en/), [United Republic of Tanzania](http://www.who.int/countries/tza/en/).

^a^The population size and the demographic age-structure were extracted from the United Nations World Population Prospects database [[1](#_ENREF_1)].

^b^HAQ Index was extracted from the Global Burden of Disease study [[4](#_ENREF_4)].

^c^The estimate for the incidence rate was based on the average of the two estimates generated by the reported COVID-19 deaths method and the reported COVID-19 cases method.

^d^The estimate for the proportion of the population infected was based on the average of the two estimates generated by the reported COVID-19 deaths method and the reported COVID-19 cases method.

**Table S2:** Estimates for key SARS-CoV-2 epidemiologic indicators for countries and territories with a population of at least one million [[1](#_ENREF_1)] in the WHO Region of the Americas (AMRO). Classification of infection severity and criticality was per WHO infection severity classifications [[2](#_ENREF_2)]. Classification of COVID-19 death was also per WHO guidelines [[3](#_ENREF_3)].

| **Region of the Americas** | **Total population [**[**1**](#_ENREF_1)**]**^a^ | **HAQ Index [**[**4**](#_ENREF_4)**]**^b^ | **Infection acute-care bed hospitalization rate (per 1,000 infections)** | **Infection ICU bed hospitalization rate (per 1,000 infections)** | **Infection severity rate**  **(per 1,000 infections)** | **Infection criticality rate**  **(per 1,000 infections)** | **Infection fatality rate**  **(per 10,000 infections)** | **Infection detection rate (%)** | **Incidence rate since epidemic onset (per 10,000 person-weeks)**^c^ | **Proportion of the population infected (%)**^d^ |
| --- | --- | --- | --- | --- | --- | --- | --- | --- | --- | --- |
| **Country** | **Thousands** | **N (95% CI)** | **N (95% CI)** | **N (95% CI)** | **N (95% CI)** | **N (95% CI)** | **N (95% CI)** | **N (95% CI)** | **N (95% CI)** | **N (95% CI)** |
| Argentina | 45,195,777 | 68.1 (65.8-70.1) | 20.4 (19.9-20.7) | 4.8 (4.7-4.8) | 7.0 (6.9-7.1) | 2.7 (2.7-2.7) | 13.4 (12.7-14.0) | 9.2 (8.2-10.5) | 142.5 (123.1-165.1) | 45.6 (40.9-50.5) |
| Bolivia | 11,673,029 | 48.8 (43.5-54.0) | 16.6 (16.2-16.8) | 3.4 (3.4-3.4) | 5.0 (4.9-5.1) | 1.8 (1.8-1.8) | 8.9 (8.5-9.4) | 6.6 (5.4-8.1) | 101.6 (80.6-128.5) | 33.5 (27.9-39.9) |
| [Brazil](http://www.who.int/countries/bra/en/) | 212,559,409 | 63.8 (62.3-64.9) | 19.8 (19.3-20.1) | 4.3 (4.3-4.4) | 6.6 (6.5-6.7) | 2.4 (2.3-2.4) | 11.2 (10.6-11.8) | 8.6 (7.8-9.7) | 152.9 (133.4-175.2) | 48.6 (44.0-53.3) |
| [Canada](http://www.who.int/countries/can/en/) | 37,742,157 | 93.8 (92.8-94.8) | 26.8 (26.1-27.2) | 7.2 (7.1-7.3) | 10.5 (10.4-10.7) | 4.2 (4.1-4.2) | 21.3 (20.3-22.4) | 12.7 (11.6-14.1) | 35.4 (32.2-38.7) | 15.7 (14.4-17.1) |
| [Chile](http://www.who.int/countries/chl/en/) | 19,116,209 | 77.9 (72.3-83.7) | 21.9 (21.4-22.3) | 5.2 (5.1-5.2) | 7.8 (7.7-7.9) | 2.9 (2.9-2.9) | 14.3 (13.6-15.1) | 10.5 (9.0-12.5) | 123.5 (101.8-151.0) | 40.5 (34.9-46.7) |
| [Colombia](http://www.who.int/countries/col/en/) | 50,882,884 | 68.5 (65.8-70.9) | 19.1 (18.6-19.3) | 4.1 (4.0-4.1) | 6.2 (6.1-6.3) | 2.2 (2.2-2.2) | 10.5 (10.0-11.1) | 9.3 (8.2-10.6) | 152.5 (130.2-179.4) | 46.5 (41.6-51.8) |
| [Costa Rica](http://www.who.int/countries/cri/en/) | 5,094,114 | 73.7 (71.2-76.0) | 20.4 (19.9-20.8) | 4.6 (4.5-4.6) | 7.0 (6.8-7.1) | 2.5 (2.5-2.6) | 12.2 (11.6-12.8) | 10.0 (8.9-11.3) | 85.5 (74.6-97.6) | 30.5 (27.2-33.9) |
| [Cuba](http://www.who.int/countries/cub/en/) | 11,326,616 | 75.5 (73.5-77.7) | 25.4 (24.8-25.8) | 6.6 (6.5-6.6) | 9.7 (9.5-9.8) | 3.7 (3.7-3.8) | 18.8 (17.9-19.8) | 10.2 (9.2-11.6) | 1.9 (1.7-2.1) | 0.8 (0.7-0.9) |
| [Dom. Rep.](http://www.who.int/countries/dom/en/) | 10,847,904 | 61.2 (57.3-65.6) | 17.3 (16.9-17.6) | 3.5 (3.5-3.6) | 5.3 (5.2-5.4) | 1.9 (1.9-1.9) | 8.9 (8.5-9.4) | 8.3 (7.1-9.8) | 45.2 (38.4-53.0) | 17.8 (15.4-20.6) |
| [Ecuador](http://www.who.int/countries/ecu/en/) | 17,643,060 | 62.2 (59.5-64.6) | 17.3 (16.9-17.6) | 3.5 (3.5-3.5) | 5.3 (5.2-5.4) | 1.9 (1.9-1.9) | 8.9 (8.4-9.3) | 8.4 (7.4-9.6) | 122.9 (104.5-145.7) | 37.2 (33.3-41.4) |
| [El Salvador](http://www.who.int/countries/slv/en/) | 6,486,201 | 63.2 (58.9-67.2) | 17.9 (17.4-18.1) | 3.8 (3.8-3.9) | 5.7 (5.6-5.8) | 2.1 (2.1-2.1) | 10.2 (9.7-10.7) | 8.6 (7.3-10.0) | 28.9 (24.9-33.5) | 11.1 (9.7-12.8) |
| [Guatemala](http://www.who.int/countries/gtm/en/) | 17,915,567 | 51.5 (45.3-57.7) | 14.2 (13.8-14.4) | 2.5 (2.5-2.5) | 3.7 (3.6-3.7) | 1.3 (1.3-1.3) | 5.9 (5.6-6.2) | 7.0 (5.6-8.6) | 49.7 (39.9-61.7) | 18.3 (15.1-22.2) |
| Haiti | 11,402,533 | 32.1 (26.6-37.8) | 14.8 (14.4-15.0) | 2.6 (2.6-2.6) | 3.9 (3.9-4.0) | 1.3 (1.3-1.3) | 5.9 (5.6-6.2) | 4.3 (3.3-5.6) | 4.0 (3.1-5.2) | 1.6 (1.3-2.1) |
| [Honduras](http://www.who.int/countries/hnd/en/) | 9,904,608 | 46.5 (40.1-53.1) | 14.5 (14.2-14.7) | 2.5 (2.5-2.6) | 3.8 (3.8-3.9) | 1.3 (1.3-1.3) | 5.9 (5.6-6.2) | 6.3 (5.0-7.9) | 63.4 (49.5-81.2) | 23.3 (18.7-28.8) |
| [Jamaica](http://www.who.int/countries/jam/en/) | 2,961,161 | 62.0 (56.8-67.3) | 19.1 (18.6-19.4) | 4.1 (4.1-4.2) | 6.2 (6.1-6.3) | 2.3 (2.2-2.3) | 10.9 (10.4-11.5) | 8.4 (7.1-10.0) | 14.1 (11.9-16.6) | 5.8 (4.9-6.8) |
| [Mexico](http://www.who.int/countries/mex/en/) | 128,932,753 | 66.3 (64.9-67.4) | 17.7 (17.2-17.9) | 3.6 (3.5-3.6) | 5.5 (5.4-5.5) | 1.9 (1.9-1.9) | 9.0 (8.5-9.4) | 9.0 (8.1-10.1) | 176.5 (149.9-212.4) | 44.0 (40.4-47.8) |
| [Nicaragua](http://www.who.int/countries/nic/en/) | 6,624,554 | 61.2 (57.0-65.4) | 15.5 (15.2-15.8) | 2.8 (2.8-2.9) | 4.3 (4.3-4.4) | 1.5 (1.5-1.5) | 6.6 (6.3-7.0) | 8.3 (7.1-9.8) | 4.2 (3.7-4.9) | 1.7 (1.5-2.0) |
| [Panama](http://www.who.int/countries/pan/en/) | 4,314,768 | 68.3 (64.6-71.9) | 18.3 (17.8-18.6) | 3.9 (3.9-3.9) | 5.8 (5.8-5.9) | 2.1 (2.1-2.1) | 10.3 (9.8-10.8) | 9.2 (8.0-10.7) | 233.1 (188.9-292.8) | 62.6 (54.9-70.9) |
| [Paraguay](http://www.who.int/countries/pry/en/) | 7,132,530 | 56.7 (53.1-60.2) | 16.3 (15.9-16.6) | 3.2 (3.1-3.2) | 4.8 (4.7-4.9) | 1.7 (1.7-1.7) | 7.8 (7.4-8.2) | 7.7 (6.6-9.0) | 59.0 (50.3-69.0) | 22.2 (19.2-25.4) |
| [Peru](http://www.who.int/countries/per/en/) | 32,971,846 | 64.3 (59.2-69.4) | 18.6 (18.1-18.9) | 3.9 (3.9-4.0) | 6.0 (5.9-6.1) | 2.1 (2.1-2.2) | 10.2 (9.7-10.7) | 8.7 (7.4-10.4) | 223.0 (170.9-311.6) | 56.1 (48.3-64.8) |
| Puerto Rico | 2,860,840 | 82.7 (80.2-85.0) | 28.8 (28.1-29.3) | 8.2 (8.1-8.3) | 11.7 (11.6-11.9) | 4.8 (4.7-4.8) | 25.1 (23.9-26.4) | 11.2 (10.0-12.7) | 57.5 (50.7-64.8) | 21.3 (19.1-23.6) |
| [Trin. and Tob.](http://www.who.int/countries/tto/en/) | 1,399,491 | 64.3 (60.7-67.5) | 22.0 (21.5-22.4) | 5.0 (5.0-5.0) | 7.7 (7.6-7.8) | 2.8 (2.7-2.8) | 13.0 (12.4-13.7) | 8.7 (7.6-10.1) | 13.2 (11.5-15.1) | 5.4 (4.7-6.1) |
| [USA](http://www.who.int/countries/usa/en/) | 331,002,647 | 88.7 (88.0-89.4) | 25.3 (24.7-25.7) | 6.6 (6.6-6.7) | 9.7 (9.5-9.9) | 3.8 (3.8-3.8) | 19.4 (18.5-20.4) | 12.0 (11.0-13.3) | 137.7 (121.9-155.2) | 49.4 (45.2-53.6) |
| [Uruguay](http://www.who.int/countries/ury/en/) | 3,473,727 | 71.0 (68.9-73.0) | 23.2 (22.6-23.6) | 6.1 (6.0-6.2) | 8.7 (8.6-8.9) | 3.5 (3.5-3.5) | 18.5 (17.6-19.4) | 9.6 (8.6-10.9) | 9.2 (8.1-10.2) | 3.7 (3.3-4.1) |
| Venezuela | 28,435,943 | 67.8 (63.6-71.8) | 18.4 (17.9-18.6) | 3.8 (3.7-3.8) | 5.8 (5.7-5.8) | 2.0 (2.0-2.0) | 9.3 (8.8-9.7) | 9.2 (7.9-10.7) | 8.9 (7.6-10.2) | 3.6 (3.1-4.2) |
| **AMRO** | **1,017,900,328** | **73.4 (71.5-75.2)** | **21.3 (20.7-21.6)** | **5.0 (4.9-5.0)** | **7.4 (7.3-7.6)** | **2.8 (2.8-2.8)** | **13.8 (13.1-14.5)** | **9.9 (8.9-11.2)** | **131.7 (113.6-154.2)** | **41.9 (37.9-46.1)** |

CI, credible interval. Dom Rep, Dominican Republic. HAQ, Healthcare Access and Quality. N, number. [Trin. and Tob.,](http://www.who.int/countries/tto/en/) [Trinidad and Tobago](http://www.who.int/countries/tto/en/).

^a^The population size and the demographic age-structure were extracted from the United Nations World Population Prospects database [[1](#_ENREF_1)].

^b^HAQ Index was extracted from the Global Burden of Disease study [[4](#_ENREF_4)].

^c^The estimate for the incidence rate was based on the average of the two estimates generated by the reported COVID-19 deaths method and the reported COVID-19 cases method.

^d^The estimate for the proportion of the population infected was based on the average of the two estimates generated by the reported COVID-19 deaths method and the reported COVID-19 cases method.

**Table S3:** Estimates for key SARS-CoV-2 epidemiologic indicators for countries and territories with a population of at least one million [[1](#_ENREF_1)] in the WHO Eastern Mediterranean Region (EMRO). Classification of infection severity and criticality was per WHO infection severity classifications [[2](#_ENREF_2)]. Classification of COVID-19 death was also per WHO guidelines [[3](#_ENREF_3)].

| **Eastern Mediterranean Region** | **Total population [**[**1**](#_ENREF_1)**]**^a^ | **HAQ Index [**[**4**](#_ENREF_4)**]**^b^ | **Infection acute-care bed hospitalization rate (per 1,000 infections)** | **Infection ICU bed hospitalization rate (per 1,000 infections)** | **Infection severity rate**  **(per 1,000 infections)** | **Infection criticality rate**  **(per 1,000 infections)** | **Infection fatality rate**  **(per 10,000 infections)** | **Infection detection rate (%)** | **Incidence rate since epidemic onset (per 10,000 person-weeks)**^c^ | **Proportion of the population infected (%)**^d^ |
| --- | --- | --- | --- | --- | --- | --- | --- | --- | --- | --- |
| **Country** | **Thousands** | **N (95% CI)** | **N (95% CI)** | **N (95% CI)** | **N (95% CI)** | **N (95% CI)** | **N (95% CI)** | **N (95% CI)** | **N (95% CI)** | **N (95% CI)** |
| [Afghanistan](http://www.who.int/countries/afg/en/) | 38,928,341 | 25.9 (22.0-29.5) | 12.0 (11.7-12.2) | 1.7 (1.6-1.7) | 2.4 (2.4-2.5) | 0.7 (0.7-0.8) | 3.0 (2.9-3.2) | 3.5 (2.7-4.4) | 10.6 (8.4-13.3) | 4.6 (3.7-5.7) |
| [Bahrain](http://www.who.int/countries/bhr/en/) | 1,701,583 | 72.0 (67.3-76.5) | 14.2 (13.9-14.4) | 2.1 (2.0-2.1) | 3.6 (3.5-3.6) | 1.0 (1.0-1.0) | 3.5 (3.3-3.7) | 9.7 (8.4-11.4) | 161.5 (131.1-201.2) | 51.0 (44.0-58.6) |
| Egypt | 102,334,403 | 58.0 (53.9-62.5) | 15.4 (15.0-15.6) | 2.7 (2.7-2.8) | 4.2 (4.1-4.3) | 1.4 (1.4-1.4) | 6.1 (5.8-6.4) | 7.8 (6.7-9.3) | 10.7 (9.2-12.4) | 4.7 (4.1-5.5) |
| Iran | 83,992,953 | 71.8 (67.3-76.3) | 17.4 (16.9-17.6) | 3.3 (3.3-3.3) | 5.2 (5.1-5.3) | 1.7 (1.7-1.7) | 7.6 (7.2-8.0) | 9.7 (8.4-11.4) | 139.8 (113.5-177.0) | 40.8 (35.8-46.4) |
| Iraq | 40,222,503 | 51.1 (45.9-56.6) | 12.9 (12.6-13.1) | 1.9 (1.9-2.0) | 2.9 (2.9-3.0) | 0.9 (0.9-0.9) | 3.8 (3.6-4.0) | 6.9 (5.7-8.4) | 97.8 (78.0-123.9) | 34.0 (28.4-40.5) |
| Jordan | 10,203,140 | 70.2 (64.8-75.3) | 14.0 (13.7-14.2) | 2.3 (2.3-2.3) | 3.5 (3.4-3.6) | 1.1 (1.1-1.1) | 4.8 (4.5-5.0) | 9.5 (8.1-11.2) | 145.1 (117.4-181.4) | 44.8 (38.6-51.7) |
| Kuwait | 4,270,563 | 80.7 (75.5-86.1) | 16.3 (15.9-16.5) | 2.5 (2.5-2.5) | 4.4 (4.3-4.5) | 1.2 (1.2-1.2) | 4.1 (3.8-4.3) | 10.9 (9.4-12.8) | 115.5 (95.9-139.8) | 39.7 (34.3-45.6) |
| Lebanon | 6,825,442 | 85.6 (82.8-88.2) | 17.7 (17.3-18.0) | 3.6 (3.5-3.6) | 5.5 (5.4-5.6) | 1.9 (1.9-1.9) | 8.8 (8.4-9.3) | 11.6 (10.3-13.2) | 56.9 (50.1-64.2) | 22.5 (20.1-25.0) |
| [Libya](http://www.who.int/countries/lby/en/) | 6,871,287 | 71.7 (67.4-74.6) | 15.2 (14.8-15.4) | 2.6 (2.6-2.6) | 4.1 (4.0-4.1) | 1.3 (1.3-1.3) | 5.4 (5.2-5.7) | 9.7 (8.4-11.1) | 66.0 (56.9-75.9) | 22.9 (20.1-25.8) |
| [Morocco](http://www.who.int/countries/mar/en/) | 36,910,558 | 57.6 (54.5-60.8) | 18.1 (17.6-18.4) | 3.6 (3.5-3.6) | 5.6 (5.5-5.7) | 1.9 (1.9-1.9) | 8.6 (8.2-9.1) | 7.8 (6.8-9.1) | 37.3 (32.2-43.0) | 15.0 (13.1-17.0) |
| [Oman](http://www.who.int/countries/omn/en/) | 5,106,622 | 76.2 (74.0-78.6) | 13.1 (12.8-13.3) | 1.8 (1.8-1.9) | 3.1 (3.0-3.1) | 0.8 (0.8-0.9) | 3.2 (3.0-3.4) | 10.3 (9.2-11.7) | 184.5 (155.1-227.2) | 49.4 (44.7-54.5) |
| [Pakistan](http://www.who.int/countries/pak/en/) | 220,892,331 | 37.6 (33.7-41.9) | 14.2 (13.9-14.4) | 2.4 (2.3-2.4) | 3.6 (3.5-3.7) | 1.2 (1.2-1.2) | 5.1 (4.9-5.4) | 5.1 (4.2-6.2) | 9.2 (7.6-11.1) | 4.0 (3.3-4.8) |
| Palestine | 5,101,416 | 57.4 (54.1-60.6) | 12.8 (12.5-13.0) | 1.9 (1.9-1.9) | 2.9 (2.8-2.9) | 0.9 (0.9-0.9) | 3.8 (3.6-4.0) | 7.8 (6.7-9.0) | 120.2 (101.1-143.2) | 40.1 (35.1-45.7) |
| [Qatar](http://www.who.int/countries/qat/en/) | 2,760,170 | 81.7 (75.9-86.6) | 12.3 (12.0-12.4) | 1.5 (1.4-1.5) | 2.6 (2.6-2.7) | 0.6 (0.6-0.6) | 1.9 (1.7-2.0) | 11.1 (9.5-12.9) | 138.5 (113.7-169.9) | 45.4 (39.2-52.3) |
| [Saudi Arabia](http://www.who.int/countries/sau/en/) | 34,813,867 | 77.1 (74.9-79.3) | 14.9 (14.5-15.1) | 2.3 (2.3-2.3) | 3.8 (3.8-3.9) | 1.1 (1.1-1.1) | 4.3 (4.0-4.5) | 10.4 (9.3-11.8) | 62.2 (55.2-70.3) | 22.6 (20.5-25.0) |
| [Somalia](http://www.who.int/countries/som/en/) | 15,893,219 | 19.0 (14.3-23.7) | 12.1 (11.8-12.3) | 1.7 (1.7-1.7) | 2.5 (2.4-2.5) | 0.8 (0.8-0.8) | 3.2 (3.1-3.4) | 2.6 (1.8-3.5) | 2.0 (1.5-2.9) | 0.8 (0.6-1.2) |
| Sudan | 43,849,269 | 45.8 (41.0-50.0) | 13.2 (12.9-13.4) | 2.0 (2.0-2.1) | 3.1 (3.0-3.1) | 1.0 (1.0-1.0) | 4.2 (4.0-4.4) | 6.2 (5.1-7.5) | 6.0 (5.0-7.1) | 2.5 (2.1-2.9) |
| [Syria](http://www.who.int/countries/syr/en/) | 17,500,657 | 67.2 (64.4-70.2) | 14.8 (14.5-15.1) | 2.5 (2.5-2.6) | 3.9 (3.9-4.0) | 1.3 (1.3-1.3) | 5.6 (5.3-5.9) | 9.1 (8.0-10.5) | 7.7 (6.9-8.7) | 3.0 (2.7-3.4) |
| [Tunisia](http://www.who.int/countries/tun/en/) | 11,818,618 | 69.4 (65.4-73.7) | 19.3 (18.9-19.6) | 4.1 (4.0-4.1) | 6.3 (6.2-6.4) | 2.2 (2.2-2.2) | 10.2 (9.7-10.7) | 9.4 (8.1-11.0) | 55.1 (47.4-64.2) | 20.8 (18.2-23.7) |
| [UAE](http://www.who.int/countries/are/en/) | 9,890,400 | 70.3 (65.5-75.4) | 13.0 (12.7-13.2) | 1.6 (1.6-1.7) | 3.0 (2.9-3.0) | 0.7 (0.7-0.7) | 2.2 (2.0-2.3) | 9.5 (8.2-11.2) | 54.0 (45.5-64.0) | 22.9 (19.7-26.5) |
| [Yemen](http://www.who.int/countries/yem/en/) | 29,825,968 | 43.3 (38.3-47.9) | 12.4 (12.0-12.5) | 1.8 (1.7-1.8) | 2.6 (2.6-2.7) | 0.8 (0.8-0.8) | 3.3 (3.2-3.5) | 5.9 (4.8-7.1) | 4.2 (3.4-4.9) | 1.5 (1.3-1.8) |
| **EMRO** | **729,713,310** | **51.5 (47.5-55.7)** | **14.7 (14.3-14.9)** | **2.5 (2.5-2.5)** | **3.8 (3.8-3.9)** | **1.2 (1.2-1.3)** | **5.4 (5.1-5.6)** | **7.0 (5.9-8.3)** | **40.5 (33.4-49.7)** | **13.7 (11.8-15.8)** |

CI, credible interval. HAQ, Healthcare Access and Quality. N, number. Syria, [Syrian Arab Republic](http://www.who.int/countries/syr/en/). UAE, [United Arab Emirates](http://www.who.int/countries/are/en/).

^a^The population size and the demographic age-structure were extracted from the United Nations World Population Prospects database [[1](#_ENREF_1)].

^b^HAQ Index was extracted from the Global Burden of Disease study [[4](#_ENREF_4)].

^c^The estimate for the incidence rate was based on the average of the two estimates generated by the reported COVID-19 deaths method and the reported COVID-19 cases method.

^d^The estimate for the proportion of the population infected was based on the average of the two estimates generated by the reported COVID-19 deaths method and the reported COVID-19 cases method.

**Table S4:** Estimates for key SARS-CoV-2 epidemiologic indicators for countries and territories with a population of at least one million [[1](#_ENREF_1)] in the WHO European Region (EURO). Classification of infection severity and criticality was per WHO infection severity classifications [[2](#_ENREF_2)]. Classification of COVID-19 death was also per WHO guidelines [[3](#_ENREF_3)].

| **European Region** | **Total population [**[**1**](#_ENREF_1)**]**^a^ | **HAQ Index [**[**4**](#_ENREF_4)**]**^b^ | **Infection acute-care bed hospitalization rate (per 1,000 infections)** | **Infection ICU bed hospitalization rate (per 1,000 infections)** | **Infection severity rate**  **(per 1,000 infections)** | **Infection criticality rate**  **(per 1,000 infections)** | **Infection fatality rate**  **(per 10,000 infections)** | **Infection detection rate (%)** | **Incidence rate since epidemic onset (per 10,000 person-weeks)**^c^ | **Proportion of the population infected (%)**^d^ |
| --- | --- | --- | --- | --- | --- | --- | --- | --- | --- | --- |
| **Country** | **Thousands** | **N (95% CI)** | **N (95% CI)** | **N (95% CI)** | **N (95% CI)** | **N (95% CI)** | **N (95% CI)** | **N (95% CI)** | **N (95% CI)** | **N (95% CI)** |
| Albania | 2,877,800 | 63.6 (75.5-78.2) | 24.5 (23.9-24.9) | 6.1 (6.0-6.2) | 9.1 (9.0-9.3) | 3.5 (3.4-3.5) | 17.1 (16.2-17.9) | 8.6 (9.4-11.7) | 52.9 (46.6-57.6) | 20.0 (17.9-21.7) |
| Armenia | 2,963,234 | 70.7 (67.8-73.5) | 22.0 (21.4-22.3) | 5.1 (5.1-5.2) | 7.8 (7.7-7.9) | 2.9 (2.9-2.9) | 14.2 (13.6-15.0) | 9.6 (8.4-11.0) | 175.4 (147.2-210.1) | 53.4 (47.3-59.8) |
| Austria | 9,006,400 | 93.9 (92.6-95.3) | 27.7 (27.0-28.1) | 7.8 (7.7-7.8) | 11.1 (11.0-11.3) | 4.5 (4.5-4.6) | 23.9 (22.7-25.1) | 12.7 (11.5-14.2) | 81.3 (72.6-90.5) | 30.1 (27.4-32.9) |
| [Azerbaijan](http://www.who.int/countries/aze/en/) | 10,139,175 | 65.6 (61.2-69.6) | 18.2 (17.7-18.5) | 3.5 (3.5-3.5) | 5.6 (5.5-5.7) | 1.8 (1.8-1.9) | 8.1 (7.7-8.5) | 8.9 (7.6-10.4) | 61.5 (52.1-72.3) | 23.6 (20.4-27.1) |
| [Belarus](http://www.who.int/countries/blr/en/) | 9,449,321 | 79.0 (75.3-82.8) | 25.0 (24.4-25.4) | 6.2 (6.2-6.3) | 9.4 (9.3-9.6) | 3.6 (3.5-3.6) | 17.9 (17.0-18.8) | 10.7 (9.4-12.4) | 32.9 (28.4-37.8) | 13.2 (11.5-14.9) |
| [Belgium](http://www.who.int/countries/bel/en/) | 11,589,616 | 92.9 (90.7-95.0) | 26.9 (26.3-27.4) | 7.6 (7.5-7.6) | 10.8 (10.6-11.0) | 4.4 (4.4-4.5) | 23.5 (22.4-24.7) | 12.6 (11.3-14.2) | 193.6 (165.5-229.3) | 57.8 (52.5-63.4) |
| [Bosnia & Herz.](http://www.who.int/countries/bih/en/) | 3,280,815 | 72.2 (67.2-76.4) | 27.3 (26.6-27.7) | 7.0 (7.0-7.1) | 10.6 (10.4-10.8) | 4.0 (4.0-4.1) | 20.1 (19.1-21.1) | 9.8 (8.4-11.4) | 126.1 (104.9-151.7) | 41.5 (36.0-47.4) |
| [Bulgaria](http://www.who.int/countries/bgr/en/) | 6,948,445 | 77.2 (73.3-80.7) | 29.5 (28.8-30.0) | 8.3 (8.2-8.3) | 12.0 (11.8-12.2) | 4.8 (4.8-4.8) | 24.7 (23.5-26.0) | 10.4 (9.1-12.0) | 92.4 (79.4-107.3) | 32.4 (28.5-36.5) |
| [Croatia](http://www.who.int/countries/hrv/en/) | 4,105,268 | 86.9 (84.5-89.4) | 28.7 (28.0-29.2) | 8.1 (8.0-8.2) | 11.7 (11.5-11.9) | 4.8 (4.7-4.8) | 25.0 (23.8-26.2) | 11.8 (10.5-13.3) | 116.1 (100.8-133.3) | 40.0 (35.8-44.3) |
| [Cyprus](http://www.who.int/countries/cyp/en/) | 1,207,361 | 90.3 (88.8-91.8) | 23.6 (23.0-24.0) | 5.9 (5.9-6.0) | 8.8 (8.6-8.9) | 3.4 (3.3-3.4) | 16.9 (16.1-17.8) | 12.2 (11.1-13.7) | 26.2 (23.4-29.0) | 10.4 (9.3-11.4) |
| [Czech Republic](http://www.who.int/countries/cze/en/) | 10,708,982 | 89.0 (87.5-90.4) | 28.3 (27.6-28.8) | 7.7 (7.7-7.8) | 11.3 (11.1-11.5) | 4.5 (4.4-4.5) | 22.8 (21.7-23.9) | 12.0 (10.9-13.5) | 164.8 (143.0-189.6) | 50.9 (46.1-55.7) |
| [Denmark](http://www.who.int/countries/dnk/en/) | 5,792,203 | 92.1 (89.8-94.3) | 28.2 (27.5-28.7) | 8.0 (7.9-8.0) | 11.4 (11.2-11.5) | 4.6 (4.6-4.7) | 24.0 (22.9-25.3) | 12.5 (11.2-14.1) | 39.7 (35.0-44.5) | 15.8 (14.1-17.5) |
| [Estonia](http://www.who.int/countries/est/en/) | 1,326,539 | 85.9 (83.6-88.3) | 27.4 (26.7-27.8) | 7.7 (7.7-7.8) | 11.1 (10.9-11.3) | 4.5 (4.5-4.6) | 24.2 (23.1-25.4) | 11.6 (10.4-13.2) | 30.7 (27.1-34.5) | 12.5 (11.1-13.8) |
| [Finland](http://www.who.int/countries/fin/en/) | 5,540,718 | 95.9 (94.6-96.9) | 29.4 (28.7-29.9) | 8.5 (8.5-8.6) | 12.1 (12.0-12.3) | 5.0 (5.0-5.0) | 26.5 (25.2-27.8) | 13.0 (11.8-14.5) | 9.6 (8.7-10.4) | 4.5 (4.1-4.9) |
| [France](http://www.who.int/countries/fra/en/) | 65,273,512 | 91.7 (90.3-93.1) | 27.6 (26.9-28.0) | 8.0 (7.9-8.0) | 11.2 (11.1-11.4) | 4.7 (4.6-4.7) | 25.1 (24.0-26.4) | 12.4 (11.3-13.9) | 89.1 (79.5-99.6) | 35.2 (32.1-38.4) |
| [Georgia](http://www.who.int/countries/geo/en/) | 3,989,175 | 67.1 (62.7-71.0) | 24.4 (23.8-24.8) | 6.2 (6.1-6.2) | 9.2 (9.0-9.3) | 3.5 (3.5-3.6) | 17.8 (17.0-18.7) | 9.1 (7.8-10.6) | 146.0 (116.8-187.4) | 44.4 (38.3-51.1) |
| [Germany](http://www.who.int/countries/deu/en/) | 83,783,945 | 92.0 (90.4-93.6) | 28.9 (28.2-29.4) | 8.5 (8.4-8.6) | 12.0 (11.8-12.2) | 5.0 (5.0-5.1) | 27.2 (25.9-28.5) | 12.4 (11.3-14.0) | 35.1 (31.5-38.8) | 15.6 (14.1-17.1) |
| [Greece](http://www.who.int/countries/grc/en/) | 10,423,056 | 90.4 (88.8-91.9) | 28.9 (28.1-29.3) | 8.7 (8.6-8.7) | 12.1 (11.9-12.3) | 5.1 (5.1-5.2) | 28.1 (26.8-29.5) | 12.2 (11.1-13.7) | 33.2 (30.0-36.5) | 13.5 (12.3-14.8) |
| [Hungary](http://www.who.int/countries/hun/en/) | 9,660,350 | 82.1 (79.5-84.9) | 28.1 (27.4-28.6) | 7.6 (7.6-7.7) | 11.2 (11.0-11.4) | 4.4 (4.4-4.5) | 22.6 (21.5-23.7) | 11.1 (9.9-12.7) | 99.4 (86.9-113.7) | 34.6 (31.0-38.5) |
| [Ireland](http://www.who.int/countries/irl/en/) | 4,937,796 | 94.6 (91.8-96.8) | 23.7 (23.1-24.1) | 6.0 (5.9-6.0) | 8.8 (8.7-8.9) | 3.4 (3.3-3.4) | 16.9 (16.1-17.8) | 12.8 (11.4-14.4) | 54.9 (48.9-61.3) | 21.0 (18.9-23.1) |
| [Israel](http://www.who.int/countries/isr/en/) | 8,655,541 | 84.8 (80.7-88.4) | 20.7 (20.2-21.0) | 5.0 (4.9-5.0) | 7.2 (7.1-7.4) | 2.8 (2.8-2.8) | 14.3 (13.6-15.1) | 11.5 (10.1-13.2) | 91.9 (78.2-107.9) | 33.2 (29.2-37.6) |
| [Italy](http://www.who.int/countries/ita/en/) | 60,461,828 | 94.9 (93.4-96.0) | 30.0 (29.2-30.5) | 9.0 (9.0-9.1) | 12.6 (12.5-12.8) | 5.4 (5.3-5.4) | 29.1 (27.8-30.6) | 12.8 (11.6-14.3) | 90.8 (81.3-101.2) | 34.9 (31.9-38.0) |
| [Kazakhstan](http://www.who.int/countries/kaz/en/) | 18,776,707 | 69.1 (64.7-73.2) | 18.3 (17.8-18.5) | 3.7 (3.6-3.7) | 5.7 (5.6-5.8) | 2.0 (2.0-2.0) | 9.1 (8.6-9.6) | 9.3 (8.1-10.9) | 30.2 (26.0-35.0) | 11.9 (10.3-13.6) |
| [Kyrgyzstan](http://www.who.int/countries/kgz/en/) | 6,524,191 | 60.6 (58.3-62.8) | 15.3 (14.9-15.5) | 2.6 (2.6-2.6) | 4.1 (4.0-4.2) | 1.3 (1.3-1.3) | 5.6 (5.3-5.8) | 8.2 (7.3-9.4) | 54.9 (48.3-62.2) | 20.1 (17.9-22.4) |
| [Latvia](http://www.who.int/countries/lva/en/) | 1,886,202 | 80.7 (78.0-83.3) | 28.3 (27.6-28.7) | 8.0 (7.9-8.0) | 11.5 (11.3-11.6) | 4.7 (4.6-4.7) | 24.5 (23.4-25.8) | 10.9 (9.7-12.4) | 39.0 (34.3-44.0) | 15.5 (13.8-17.3) |
| [Lithuania](http://www.who.int/countries/ltu/en/) | 2,722,291 | 80.5 (78.7-82.3) | 28.1 (27.4-28.6) | 8.0 (7.9-8.1) | 11.5 (11.3-11.6) | 4.7 (4.7-4.7) | 25.1 (24.0-26.4) | 10.9 (9.8-12.3) | 97.1 (84.0-111.5) | 33.1 (29.7-36.6) |
| [Netherlands](http://www.who.int/countries/nld/en/) | 17,134,873 | 96.1 (94.5-97.3) | 28.2 (27.5-28.7) | 7.9 (7.8-7.9) | 11.3 (11.2-11.5) | 4.6 (4.5-4.6) | 23.7 (22.6-24.9) | 13.0 (11.8-14.5) | 89.3 (79.4-99.9) | 32.3 (29.4-35.4) |
| [Norway](http://www.who.int/countries/nor/en/) | 5,421,242 | 96.6 (94.9-97.9) | 26.0 (25.3-26.4) | 7.0 (6.9-7.1) | 10.1 (10.0-10.3) | 4.0 (4.0-4.1) | 20.8 (19.8-21.9) | 13.1 (11.8-14.6) | 12.8 (11.5-14.1) | 5.5 (5.0-6.0) |
| [Poland](http://www.who.int/countries/pol/en/) | 37,846,605 | 82.4 (79.7-84.6) | 27.1 (26.4-27.5) | 7.2 (7.2-7.3) | 10.7 (10.5-10.8) | 4.2 (4.2-4.2) | 21.6 (20.5-22.7) | 11.1 (9.9-12.6) | 86.5 (75.9-98.2) | 31.1 (27.9-34.5) |
| [Portugal](http://www.who.int/countries/prt/en/) | 10,196,707 | 85.7 (84.1-87.3) | 29.7 (29.0-30.2) | 8.8 (8.7-8.9) | 12.4 (12.2-12.6) | 5.2 (5.1-5.2) | 27.8 (26.5-29.2) | 11.6 (10.5-13.0) | 78.1 (69.1-87.7) | 28.5 (25.8-31.3) |
| [Rep. Moldova](http://www.who.int/countries/mda/en/) | 4,033,963 | 67.4 (64.5-70.4) | 22.9 (22.3-23.2) | 5.2 (5.1-5.2) | 8.1 (8.0-8.2) | 2.9 (2.8-2.9) | 13.4 (12.7-14.1) | 9.1 (8.0-10.5) | 118.5 (101.3-138.7) | 39.7 (35.1-44.7) |
| [Romania](http://www.who.int/countries/rou/en/) | 19,237,682 | 78.3 (75.9-80.7) | 27.4 (26.7-27.9) | 7.4 (7.4-7.5) | 10.9 (10.7-11.0) | 4.3 (4.3-4.3) | 22.2 (21.1-23.3) | 10.6 (9.5-12.0) | 84.3 (73.9-95.8) | 31.1 (27.8-34.5) |
| [Russia](http://www.who.int/countries/rus/en/) | 145,934,460 | 75.1 (67.7-81.7) | 24.8 (24.2-25.2) | 6.2 (6.2-6.3) | 9.4 (9.2-9.5) | 3.6 (3.5-3.6) | 18.0 (17.1-18.9) | 10.2 (8.4-12.2) | 45.6 (37.6-55.3) | 19.6 (16.5-23.2) |
| Serbia | 8,737,370 | 77.2 (74.9-79.3) | 27.2 (26.5-27.7) | 7.2 (7.2-7.3) | 10.7 (10.5-10.8) | 4.2 (4.1-4.2) | 21.0 (20.0-22.1) | 10.4 (9.3-11.8) | 71.9 (62.4-82.2) | 25.7 (22.9-28.6) |
| [Slovakia](http://www.who.int/countries/svk/en/) | 5,459,643 | 83.3 (80.4-86.3) | 26.1 (25.5-26.5) | 6.6 (6.6-6.7) | 10.0 (9.8-10.1) | 3.8 (3.7-3.8) | 18.6 (17.7-19.6) | 11.3 (10.0-12.9) | 65.2 (56.8-74.4) | 24.2 (21.5-27.1) |
| [Slovenia](http://www.who.int/countries/svn/en/) | 2,078,932 | 90.8 (88.2-93.4) | 28.6 (27.8-29.0) | 8.0 (7.9-8.0) | 11.6 (11.4-11.7) | 4.7 (4.6-4.7) | 24.3 (23.1-25.5) | 12.3 (11.0-13.9) | 159.2 (137.3-184.8) | 49.6 (44.6-54.9) |
| [Spain](http://www.who.int/countries/esp/en/) | 46,754,783 | 91.9 (90.5-93.2) | 27.9 (27.2-28.3) | 8.0 (7.9-8.1) | 11.4 (11.2-11.5) | 4.7 (4.6-4.7) | 25.1 (23.9-26.3) | 12.4 (11.3-13.9) | 100.4 (89.5-112.3) | 37.7 (34.5-41.2) |
| [Sweden](http://www.who.int/countries/swe/en/) | 10,099,270 | 95.5 (93.4-97.2) | 27.7 (27.0-28.1) | 7.9 (7.8-8.0) | 11.2 (11.0-11.4) | 4.6 (4.6-4.7) | 24.5 (23.3-25.7) | 12.9 (11.6-14.5) | 90.6 (80.2-102.0) | 35.1 (31.8-38.5) |
| [Switzerland](http://www.who.int/countries/che/en/) | 8,654,618 | 95.6 (92.4-97.8) | 27.4 (26.7-27.9) | 7.7 (7.6-7.7) | 11.0 (10.8-11.2) | 4.5 (4.4-4.5) | 23.5 (22.4-24.7) | 12.9 (11.5-14.6) | 107.1 (93.3-122.5) | 37.8 (33.9-41.9) |
| [Tajikistan](http://www.who.int/countries/tjk/en/) | 9,537,642 | 51.7 (47.7-55.5) | 13.5 (13.2-13.7) | 2.0 (2.0-2.0) | 3.2 (3.1-3.2) | 1.0 (1.0-1.0) | 3.9 (3.7-4.1) | 7.0 (5.9-8.3) | 4.9 (4.2-5.7) | 1.7 (1.4-2.0) |
| [Macedonia](http://www.who.int/countries/mkd/en/) | 2,083,380 | 75.1 (72.6-77.5) | 24.5 (23.9-24.9) | 5.9 (5.9-6.0) | 9.0 (8.9-9.2) | 3.3 (3.3-3.4) | 16.0 (15.2-16.8) | 10.2 (9.0-11.6) | 161.4 (138.2-189.8) | 49.8 (44.7-55.3) |
| [Turkey](http://www.who.int/countries/tur/en/) | 84,339,067 | 74.4 (70.0-78.4) | 19.1 (18.6-19.4) | 4.1 (4.0-4.1) | 6.2 (6.1-6.3) | 2.2 (2.2-2.2) | 10.4 (9.9-11.0) | 10.1 (8.7-11.7) | 46.4 (40.0-53.7) | 17.8 (15.5-20.2) |
| [Turkmenistan](http://www.who.int/countries/tkm/en/) | 6,031,187 | 61.6 (58.7-64.8) | 15.3 (15.0-15.6) | 2.6 (2.6-2.6) | 4.1 (4.1-4.2) | 1.3 (1.3-1.3) | 5.6 (5.3-5.9) | 8.3 (7.3-9.7) | N/A^e^ | N/A^e^ |
| [Ukraine](http://www.who.int/countries/ukr/en/) | 43,733,759 | 74.6 (68.3-79.8) | 25.9 (25.3-26.3) | 6.7 (6.7-6.8) | 10.0 (9.8-10.2) | 3.9 (3.8-3.9) | 19.6 (18.7-20.6) | 10.1 (8.5-11.9) | 54.1 (45.3-64.7) | 20.8 (17.8-24.3) |
| [United Kingdom](http://www.who.int/countries/gbr/en/) | 67,886,004 | 90.5 (89.6-91.3) | 26.6 (25.9-27.0) | 7.4 (7.3-7.5) | 10.6 (10.4-10.7) | 4.3 (4.3-4.3) | 22.7 (21.6-23.9) | 12.2 (11.2-13.6) | 101.1 (90.8-112.5) | 37.7 (34.6-40.9) |
| [Uzbekistan](http://www.who.int/countries/uzb/en/) | 33,469,199 | 62.9 (59.3-66.0) | 15.5 (15.1-15.8) | 2.6 (2.6-2.7) | 4.2 (4.2-4.3) | 1.3 (1.3-1.3) | 5.6 (5.3-5.9) | 8.5 (7.4-9.8) | 6.1 (5.3-7.0) | 2.5 (2.2-2.9) |
| **EURO** | **930,700,857** | **82.6 (79.4-85.7)** | **25.3 (24.7-25.8)** | **6.7 (6.7-6.8)** | **9.8 (9.7-10.0)** | **3.9 (3.9-3.9)** | **20.2 (19.2-21.2)** | **11.2 (9.9-12.8)** | **67.4 (58.8-77.1)** | **25.5 (22.8-28.4)** |

[Bosnia & Herz,](http://www.who.int/countries/bih/en/) [Bosnia and Herzegovina](http://www.who.int/countries/bih/en/). CI, credible interval. HAQ, Healthcare Access and Quality. Macedonia, [Republic of North Macedonia](http://www.who.int/countries/mkd/en/). N, number. [Rep Moldova](http://www.who.int/countries/mda/en/), [Republic of Moldova](http://www.who.int/countries/mda/en/). Russia, [Russian Federation](http://www.who.int/countries/rus/en/).

^a^The population size and the demographic age-structure were extracted from the United Nations World Population Prospects database [[1](#_ENREF_1)].

^b^HAQ Index was extracted from the Global Burden of Disease study [[4](#_ENREF_4)].

^c^The estimate for the incidence rate was based on the average of the two estimates generated by the reported COVID-19 deaths method and the reported COVID-19 cases method.

^d^The estimate for the proportion of the population infected was based on the average of the two estimates generated by the reported COVID-19 deaths method and the reported COVID-19 cases method.

^e^No reported COVID-19 cases and deaths.

**Table S5:** Estimates for key SARS-CoV-2 epidemiologic indicators for countries and territories with a population of at least one million [[1](#_ENREF_1)] in the WHO South-East Asia Region (SEARO). Classification of infection severity and criticality was per WHO infection severity classifications [[2](#_ENREF_2)]. Classification of COVID-19 death was also per WHO guidelines [[3](#_ENREF_3)].

| **South-East Asia Region** | **Total population [**[**1**](#_ENREF_1)**]**^a^ | **HAQ Index [**[**4**](#_ENREF_4)**]**^b^ | **Infection acute-care bed hospitalization rate (per 1,000 infections)** | **Infection ICU bed hospitalization rate (per 1,000 infections)** | **Infection severity rate**  **(per 1,000 infections)** | **Infection criticality rate**  **(per 1,000 infections)** | **Infection fatality rate**  **(per 10,000 infections)** | **Infection detection rate (%)** | **Incidence rate since epidemic onset (per 10,000 person-weeks)**^c^ | **Proportion of the population infected (%)**^d^ |
| --- | --- | --- | --- | --- | --- | --- | --- | --- | --- | --- |
| **Country** | **Thousands** | **N (95% CI)** | **N (95% CI)** | **N (95% CI)** | **N (95% CI)** | **N (95% CI)** | **N (95% CI)** | **N (95% CI)** | **N (95% CI)** | **N (95% CI)** |
| [Bangladesh](http://www.who.int/countries/bgd/en/) | 164,689,383 | 47.6 (44.3-50.9) | 15.6 (15.2-15.9) | 2.9 (2.8-2.9) | 4.4 (4.3-4.4) | 1.5 (1.5-1.5) | 6.6 (6.2-6.9) | 6.4 (5.5-7.6) | 10.2 (8.7-11.9) | 4.3 (3.7-4.9) |
| [DPR. Korea](http://www.who.int/countries/prk/en/) | 25,778,815 | 53.4 (49.6-56.9) | 21.3 (20.7-21.6) | 4.7 (4.7-4.8) | 7.3 (7.2-7.4) | 2.6 (2.6-2.6) | 12.1 (11.5-12.7) | 7.2 (6.2-8.5) | N/A^e^ | N/A^e^ |
| [India](http://www.who.int/countries/ind/en/) | 1,380,004,385 | 41.2 (39.1-43.4) | 16.9 (16.5-17.2) | 3.2 (3.2-3.2) | 5.0 (4.9-5.1) | 1.7 (1.7-1.7) | 7.4 (7.0-7.8) | 5.6 (4.9-6.5) | 22.1 (19.0-25.4) | 10.0 (8.7-11.4) |
| [Indonesia](http://www.who.int/countries/idn/en/) | 273,523,621 | 44.5 (42.6-46.8) | 17.3 (16.8-17.5) | 3.2 (3.2-3.3) | 5.1 (5.0-5.2) | 1.7 (1.7-1.7) | 7.3 (6.9-7.6) | 6.0 (5.3-7.0) | 11.7 (10.3-13.3) | 5.0 (4.4-5.6) |
| [Myanmar](http://www.who.int/countries/mmr/en/) | 54,409,794 | 41.6 (38.0-45.5) | 17.1 (16.6-17.3) | 3.1 (3.1-3.2) | 5.0 (4.9-5.1) | 1.6 (1.6-1.6) | 6.9 (6.5-7.3) | 5.6 (4.7-6.8) | 9.3 (7.8-11.0) | 3.7 (3.1-4.3) |
| [Nepal](http://www.who.int/countries/npl/en/) | 29,136,808 | 40.0 (36.5-44.4) | 15.7 (15.3-15.9) | 2.9 (2.8-2.9) | 4.4 (4.3-4.4) | 1.5 (1.5-1.5) | 6.5 (6.2-6.9) | 5.4 (4.5-6.6) | 22.8 (18.6-27.6) | 10.4 (8.6-12.3) |
| [Sri Lanka](http://www.who.int/countries/lka/en/) | 21,413,250 | 70.6 (66.3-75.3) | 21.6 (21.1-22.0) | 4.9 (4.8-4.9) | 7.4 (7.3-7.6) | 2.7 (2.7-2.7) | 12.5 (11.9-13.2) | 9.6 (8.3-11.2) | 2.8 (2.4-3.2) | 1.3 (1.1-1.5) |
| [Thailand](http://www.who.int/countries/tha/en/) | 69,799,978 | 69.5 (66.5-72.6) | 23.7 (23.1-24.1) | 5.7 (5.6-5.7) | 8.6 (8.5-8.8) | 3.2 (3.1-3.2) | 15.3 (14.5-16.0) | 9.4 (8.3-10.8) | 0.1 (0.1-0.2) | 0.1 (0.1-0.1) |
| [Timor-Leste](http://www.who.int/countries/tls/en/) | 1,318,442 | 43.4 (37.2-51.9) | 13.9 (13.6-14.1) | 2.3 (2.3-2.4) | 3.5 (3.4-3.5) | 1.2 (1.1-1.2) | 5.1 (4.8-5.3) | 5.9 (4.6-7.7) | 0.1 (0.1-0.1) | 0.0 (0.0-0.0) |
| **SEARO** | **2,020,074,476** | **43.6 (41.3-46.1)** | **17.2 (16.8-17.4)** | **3.3 (3.2-3.3)** | **5.1 (5.0-5.2)** | **1.7 (1.7-1.7)** | **7.6 (7.3-8.0)** | **5.9 (5.1-6.9)** | **18.1 (15.6-20.8)** | **8.1 (7.0-9.2)** |

CI, credible interval. [DPR Korea](http://www.who.int/countries/prk/en/), [Democratic People's Republic of Korea](http://www.who.int/countries/prk/en/). HAQ, Healthcare Access and Quality. N, number.

^a^The population size and the demographic age-structure were extracted from the United Nations World Population Prospects database [[1](#_ENREF_1)].

^b^HAQ Index was extracted from the Global Burden of Disease study [[4](#_ENREF_4)].

^c^The estimate for the incidence rate was based on the average of the two estimates generated by the reported COVID-19 deaths method and the reported COVID-19 cases method.

^d^The estimate for the proportion of the population infected was based on the average of the two estimates generated by the reported COVID-19 deaths method and the reported COVID-19 cases method.

^e^No reported COVID-19 cases and deaths.

**Table S6:** Estimates for key SARS-CoV-2 epidemiologic indicators for countries and territories with a population of at least one million [[1](#_ENREF_1)] in the WHO Western Pacific Region (WPRO). Classification of infection severity and criticality was per WHO infection severity classifications [[2](#_ENREF_2)]. Classification of COVID-19 death was also per WHO guidelines [[3](#_ENREF_3)].

| **Western Pacific Region** | **Total population [**[**1**](#_ENREF_1)**]**^a^ | **HAQ Index [**[**4**](#_ENREF_4)**]**^b^ | **Infection acute-care bed hospitalization rate (per 1,000 infections)** | **Infection ICU bed hospitalization rate (per 1,000 infections)** | **Infection severity rate**  **(per 1,000 infections)** | **Infection criticality rate**  **(per 1,000 infections)** | **Infection fatality rate**  **(per 10,000 infections)** | **Infection detection rate (%)** | **Incidence rate since epidemic onset (per 10,000 person-weeks)**^c^ | **Proportion of the population infected (%)**^d^ |
| --- | --- | --- | --- | --- | --- | --- | --- | --- | --- | --- |
| **Country** | **Thousands** | **N (95% CI)** | **N (95% CI)** | **N (95% CI)** | **N (95% CI)** | **N (95% CI)** | **N (95% CI)** | **N (95% CI)** | **N (95% CI)** | **N (95% CI)** |
| [Australia](http://www.who.int/countries/aus/en/) | 25,499,881 | 95.9 (94.8-96.8) | 24.7 (24.0-25.1) | 6.5 (6.4-6.6) | 9.4 (9.3-9.6) | 3.7 (3.7-3.8) | 19.3 (18.4-20.3) | 13.0 (11.8-14.4) | 2.9 (2.7-3.1) | 1.4 (1.3-1.5) |
| [Cambodia](http://www.who.int/countries/khm/en/) | 16,718,971 | 39.4 (36.4-42.5) | 14.9 (14.6-15.2) | 2.5 (2.5-2.5) | 3.9 (3.9-4.0) | 1.3 (1.3-1.3) | 5.4 (5.1-5.6) | 5.3 (4.5-6.3) | 0.0 (0.0-0.0) | 0.0 (0.0-0.0) |
| [China](http://www.who.int/countries/chn/en/) | 1,439,323,774 | 77.9 (76.5-78.9) | 22.8 (22.2-23.1) | 5.1 (5.1-5.1) | 8.0 (7.9-8.1) | 2.8 (2.8-2.8) | 12.9 (12.3-13.6) | 10.5 (9.5-11.8) | 0.3 (0.3-0.3) | 0.1 (0.1-0.2) |
| Hong Kong | 7,496,988 | 77.9 (76.5-78.9) | 27.5 (26.8-28.0) | 7.4 (7.3-7.4) | 10.9 (10.7-11.1) | 4.3 (4.2-4.3) | 21.8 (20.8-22.9) | 10.5 (9.5-11.8) | 2.1 (1.9-2.3) | 0.9 (0.9-1.0) |
| [Japan](http://www.who.int/countries/jpn/en/) | 126,476,458 | 94.1 (93.5-94.6) | 32.6 (31.7-33.1) | 10.5 (10.4-10.6) | 14.3 (14.1-14.5) | 6.3 (6.2-6.3) | 34.9 (33.3-36.6) | 12.7 (11.7-14.1) | 2.2 (2.0-2.4) | 1.1 (1.0-1.2) |
| [Lao PDR.](http://www.who.int/countries/lao/en/) | 7,275,556 | 36.6 (32.6-41.1) | 14.3 (14.0-14.6) | 2.3 (2.3-2.4) | 3.6 (3.6-3.7) | 1.2 (1.1-1.2) | 4.9 (4.6-5.1) | 5.0 (4.1-6.1) | 0.0 (0.0-0.0) | 0.0 (0.0-0.0) |
| [Malaysia](http://www.who.int/countries/mys/en/) | 32,365,998 | 68.1 (65.9-70.2) | 17.6 (17.2-17.9) | 3.4 (3.4-3.5) | 5.4 (5.3-5.5) | 1.8 (1.8-1.8) | 8.2 (7.8-8.6) | 9.2 (8.2-10.5) | 5.2 (4.6-5.9) | 2.5 (2.2-2.8) |
| [Mongolia](http://www.who.int/countries/mng/en/) | 3,278,292 | 53.4 (49.1-57.6) | 15.3 (14.9-15.5) | 2.6 (2.5-2.6) | 4.1 (4.0-4.1) | 1.3 (1.3-1.3) | 5.2 (4.9-5.5) | 7.2 (6.1-8.6) | 0.6 (0.5-0.7) | 0.3 (0.2-0.3) |
| [New Zealand](http://www.who.int/countries/nzl/en/) | 4,822,233 | 92.4 (90.3-94.3) | 25.1 (24.5-25.5) | 6.6 (6.5-6.7) | 9.6 (9.5-9.8) | 3.8 (3.8-3.8) | 19.3 (18.4-20.3) | 12.5 (11.3-14.1) | 0.7 (0.6-0.7) | 0.3 (0.3-0.3) |
| [Pap. New Gui](http://www.who.int/countries/png/en/) | 8,947,027 | 31.8 (26.2-37.4) | 13.8 (13.4-14.0) | 2.1 (2.1-2.1) | 3.3 (3.2-3.4) | 1.0 (1.0-1.0) | 4.0 (3.8-4.2) | 4.3 (3.3-5.6) | 0.4 (0.3-0.5) | 0.1 (0.1-0.2) |
| [Philippines](http://www.who.int/countries/phl/en/) | 109,581,085 | 51.2 (47.9-54.4) | 15.7 (15.3-16.0) | 2.8 (2.8-2.8) | 4.4 (4.3-4.4) | 1.4 (1.4-1.5) | 6.3 (6.0-6.7) | 6.9 (6.0-8.1) | 14.8 (12.8-17.1) | 6.9 (5.9-7.9) |
| [Rep Korea](http://www.who.int/countries/kor/en/) | 51,269,183 | 90.3 (85.6-93.9) | 26.4 (25.8-26.9) | 6.7 (6.7-6.8) | 10.1 (10.0-10.3) | 3.8 (3.8-3.9) | 18.9 (18.0-19.9) | 12.2 (10.7-14.0) | 1.9 (1.7-2.2) | 0.9 (0.8-1.1) |
| Taiwan | 23,816,775 | 85.4 (82.5-88.2) | 25.8 (25.2-26.2) | 6.5 (6.4-6.5) | 9.8 (9.7-10.0) | 3.7 (3.7-3.7) | 18.2 (17.4-19.2) | 11.6 (10.3-13.2) | 0.0 (0.0-0.1) | 0.0 (0.0-0.0) |
| [Singapore](http://www.who.int/countries/sgp/en/) | 5,850,343 | 90.6 (87.2-93.3) | 24.8 (24.2-25.2) | 5.7 (5.6-5.7) | 9.0 (8.9-9.2) | 3.2 (3.1-3.2) | 14.6 (13.9-15.4) | 12.3 (10.9-13.9) | 9.0 (7.9-10.2) | 4.3 (3.7-4.8) |
| [Viet Nam](http://www.who.int/countries/vnm/en/) | 97,338,583 | 60.3 (56.3-64.1) | 18.4 (18.0-18.7) | 3.8 (3.7-3.8) | 5.8 (5.8-5.9) | 2.0 (2.0-2.0) | 9.5 (9.0-10.0) | 8.2 (7.0-9.6) | 0.0 (0.0-0.1) | 0.0 (0.0-0.0) |
| **WPRO** | **1,960,061,147** | **76.4 (74.7-77.8)** | **22.8 (22.2-23.1)** | **5.3 (5.2-5.3)** | **8.1 (8.0-8.2)** | **2.9 (2.9-2.9)** | **13.9 (13.2-14.6)** | **10.3 (9.3-11.6)** | **1.4 (1.2-1.6)** | **0.7 (0.6-0.7)** |

CI, credible interval. HAQ, Healthcare Access and Quality. Lao PDR, [Lao People's Democratic Republic](http://www.who.int/countries/lao/en/). N, number. [Pap New Gui](http://www.who.int/countries/png/en/), [Papua New Guinea](http://www.who.int/countries/png/en/). [Rep Korea](http://www.who.int/countries/kor/en/), [Republic of Korea](http://www.who.int/countries/kor/en/).

^a^The population size and the demographic age-structure were extracted from the United Nations World Population Prospects database [[1](#_ENREF_1)].

^b^HAQ Index was extracted from the Global Burden of Disease study [[4](#_ENREF_4)].

^c^The estimate for the incidence rate was based on the average of the two estimates generated by the reported COVID-19 deaths method and the reported COVID-19 cases method.

^d^The estimate for the proportion of the population infected was based on the average of the two estimates generated by the reported COVID-19 deaths method and the reported COVID-19 cases method.

**Figure S1: Sensitivity analysis for the application of the Global Burden of Disease study’s Healthcare Access and Quality (HAQ) Index ratio.** Estimated percentages of populations infected across WHO regions and globally as of the end of 2020. A) With no application of the HAQ ratio compared to baseline analysis. B) Adjustment using the HAQ ratio to power of ½ (square root of HAQ ratio) compared to baseline analysis. C) Adjustment using the HAQ ratio to power of 2 (square of HAQ ratio) compared to baseline analysis. The analysis was generated using the reported COVID-19 cases method. WHO regions include the African Region (AFRO), the Eastern Mediterranean Region (EMRO), the South-East Asia Region (SEARO), the Region of the Americas (AMRO), the Western Pacific Region (WPRO), and the European Region (EURO) (Figure 1 of main text).


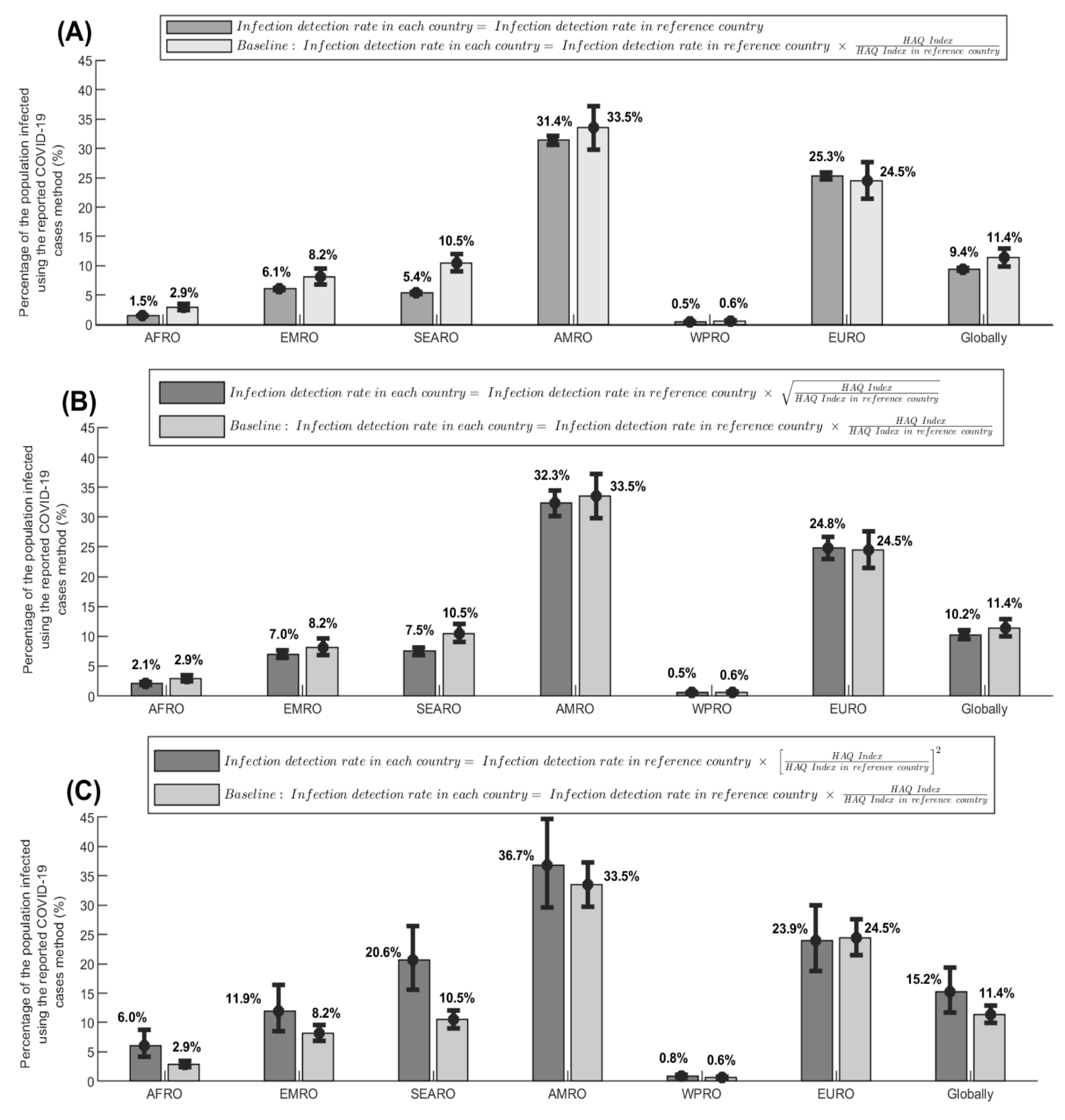


**Figure S2: Sensitivity analysis applying a different input estimate for the infection detection rate, that for the United States (US) [**[**5**](#_ENREF_5)**] instead of that for Qatar, as the reference country.** Estimated percentages of populations infected across WHO regions and globally as of the end of 2020 in the sensitivity analysis compared to the baseline analysis. The analysis was generated using the reported COVID-19 cases method. WHO regions include the African Region (AFRO), the Eastern Mediterranean Region (EMRO), the South-East Asia Region (SEARO), the Region of the Americas (AMRO), the Western Pacific Region (WPRO), and the European Region (EURO) (Figure 1 of main text).


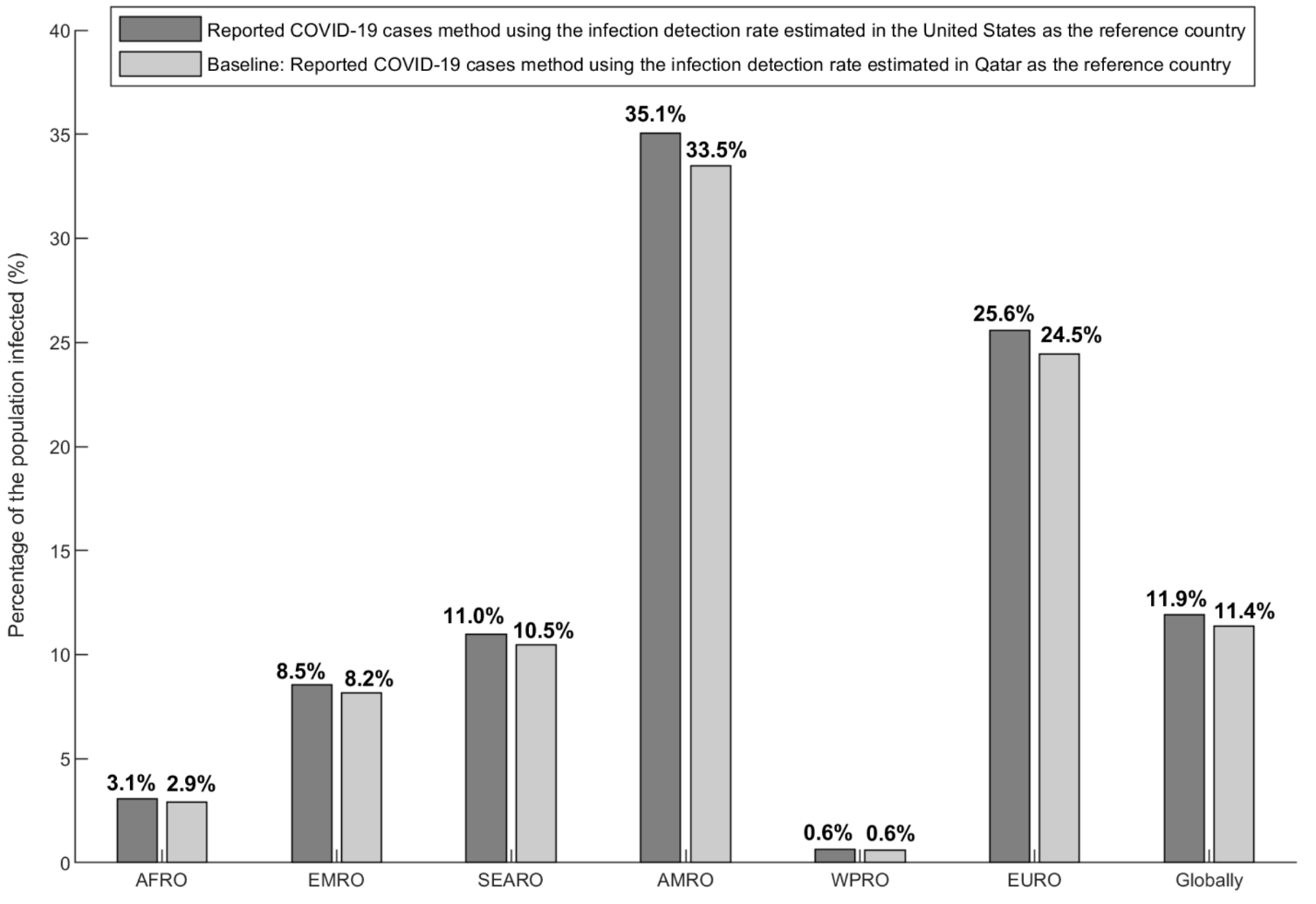


**References**

1. United Nations Department of Economic and Social Affairs Population Dynamics, *The 2019 Revision of World Population Prospects. Available from* [*https://population.un.org/wpp/*](https://population.un.org/wpp/)*. Accessed on March 1st, 2020.* 2020.

2. World Health Organization, *Clinical management of COVID-19. Available from:* [*https://www.who.int/publications-detail/clinical-management-of-covid-19*](https://www.who.int/publications-detail/clinical-management-of-covid-19)*. Accessed on: May 31st 2020.* 2020.

3. World Health Organization, *International guidelines for certification and classification (coding) of COVID-19 as cause of death. Available from:* [*https://www.who.int/classifications/icd/Guidelines_Cause_of_Death_COVID-19-20200420-EN.pdf?ua=1*](https://www.who.int/classifications/icd/Guidelines_Cause_of_Death_COVID-19-20200420-EN.pdf?ua=1)*. Document Number: WHO/HQ/DDI/DNA/CAT. Accessed on June 1, 2020. .* 2020.

4. Fullman, N., et al., *Measuring performance on the Healthcare Access and Quality Index for 195 countries and territories and selected subnational locations: a systematic analysis from the Global Burden of Disease Study 2016.* The Lancet, 2018. **391**(10136): p. 2236-2271.

5. Angulo, F.J., L. Finelli, and D.L. Swerdlow, *Estimation of US SARS-CoV-2 Infections, Symptomatic Infections, Hospitalizations, and Deaths Using Seroprevalence Surveys.* JAMA Netw Open, 2021. **4**(1): p. e2033706.

6. Seedat, S., et al., *SARS-CoV-2 infection hospitalization, severity, criticality, and fatality rates in Qatar.* Sci Rep, 2021. **11**(1): p. 18182.
